# Paecilins F–P, new dimeric chromanones isolated from the endophytic fungus *Xylaria curta* E10, and structural revision of paecilin A

**DOI:** 10.3389/fmicb.2022.922444

**Published:** 2022-09-02

**Authors:** Pan-Pan Wei, Hong-Lian Ai, Bao-Bao Shi, Ke Ye, Xiao Lv, Xiao-Yan Pan, Xu-Jun Ma, Dan Xiao, Zheng-Hui Li, Xin-Xiang Lei

**Affiliations:** School of Pharmaceutical Sciences, South-Central University for Nationalities, Wuhan, China

**Keywords:** dimeric chromanones, endophytic fungus, *Xylaria curta* E10, antimicrobial activities, structural identification

## Abstract

A total of eleven new dimeric chromanones, paecilins F-P (**2**–**12**), were isolated from the endophytic fungus *Xylaria curta* E10, along with four known analogs (**1**, **13**–**15**). Their structures and absolute configurations were determined by extensive experimental spectroscopic methods, single-crystal X-ray diffraction, and equivalent circulating density (ECD) calculations. In addition, the structure of paecilin A, which was reported to be a symmetric C8-C8′ dimeric pattern, was revised by analysis of the nuclear magnetic resonance (NMR) data, and single-crystal X-ray diffraction. Compound **1** showed antifungal activity against the human pathogenic fungus *Candida albicans* with a minimum inhibitory concentration of 16 μg/mL, and Compounds **8** and **10** showed antibacterial activity against the gram-negative bacterium *Escherichia coli* with the same minimum inhibitory concentration of 16 μg/mL.

## Introduction

Chromanones are a class of compounds with benzo-γ-pyranone skeletons. They are widely distributed in plants, fungi, and lichens (Duan et al., 2019). Chromanones usually form homodimers or heterodimers with xanthones, the biosynthetic precursors of chromanones, with different linkages, including C6-C6, C6-C8, and C8-C8 (Wu et al., 2015; Cao et al., 2022). To unambiguously determine the absolute or even relative configurations of these dimers remains challenging because of the presence of various chiral centers, sometimes with axial chirality. These compounds have been reported to have good biological activities, such as antitumor (Pinto et al., 2014; El-Elimat et al., 2015; Wu et al., 2015; Arora et al., 2017; Nguyen et al., 2020; Tuong et al., 2020; Lünne et al., 2021; Wang et al., 2021), antifungal (da Silva et al., 2018) and antibacterial (Pontius et al., 2008; Kumla et al., 2017; Wang et al., 2021) activities. Their dimers have attracted much attention from scientists in the fields of chemical synthesis and biosynthesis for many years because of their complex structures and remarkable biological activity (Xiao et al., 2017; Wei and Matsuda, 2020; Wei et al., 2021).

Endophytic fungi are microorganisms that live in healthy plant tissues and do not cause any loss or disease to the host plant (Ribeiro et al., 2021). Endophytic fungi are an important resource of natural bioactive compounds (Li et al., 2008; Liu et al., 2014; Chen et al., 2016; Nalin Rathnayake et al., 2019). For many years, they have attracted much attention because of their ability to produce new bioactive secondary metabolites (Liu et al., 2019). *Xylaria curta* E10 is an endophytic fungus isolated from *Solanum tuberosum*. A previous investigation of this fungus led to the isolation of several cytochalasans with novel scaffolds and intriguing biological activity. Curtachalasins A and B are two cytochalasans with 5/6/6/6 tetracyclic skeletons (Wang et al., 2018), curtachalasins C-E have an unprecedented bridged 6/6/6/6 ring system with significant resistance reversal activity against fluconazole-resistant *Candida albicans* (Wang et al., 2019a), and xylarichalasin A possesses a 6/7/5/6/6/6 fused polycyclic system with remarkable anti-proliferative activity (Wang et al., 2019b).

**FIGURE 1:**
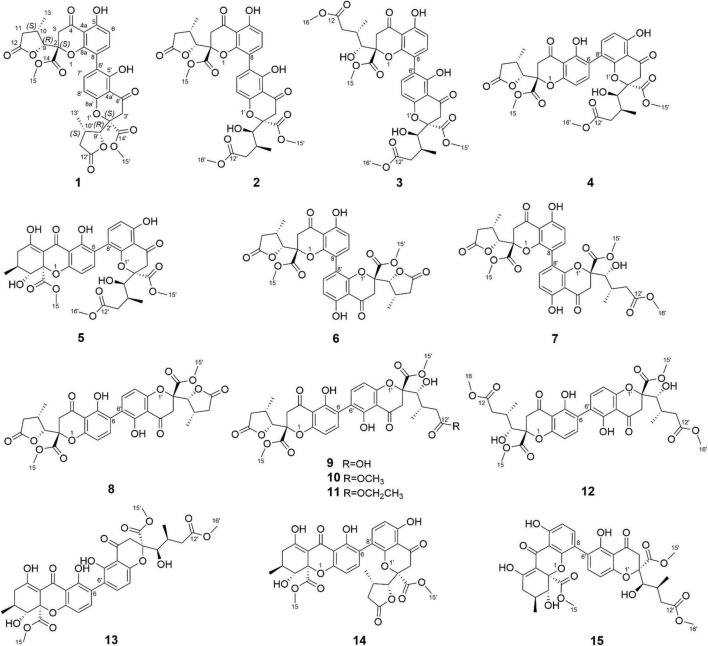
Structures of Compounds **1**–**15**.

In our ongoing research on mining structurally interesting and biologically active constituents from natural resources, a chemical study on the endophytic fungus *Xylaria curta* E10 was carried out. As a result, eleven new dimeric chromanones named paecilins F-Q (**2**–**12**), together with four known compounds, paecilin A (**1**) (Guo et al., 2007), versixanthone F (**13**) (Wu et al., 2015), versixanthone A (**14**) (Wu et al., 2015), and versixanthone E (**15**) (Wu et al., 2015) (Figure 1) were isolated. Herein, the details of the isolation, structural elucidation, and antimicrobial activities of these compounds are presented.

## Materials and methods

### Fungal material

The strain required in this study was isolated from fresh healthy potato tissues collected from Dali City, Yunnan Province, China, and identified as *Xylaria curta* E10 according to the ITS sequence (GenBank Accession No. KJ883611.1, query cover 100%, maximum identity 99%). At present, the strain is stored in the microbial seed bank of the School of Pharmacy, South-Central University for Nationalities. The fungus *Xylaria curta* E10 was fermented in a solid rice medium (100 g of rice and 100 ml of water, in each 500 ml culture flask, with a total of 15 kg of rice) and was cultured at 24°C for one month.

### Fungal fermentation, extraction, and isolation

The fermented materials were soaked in absolute MeOH (20 L × 5). The combined extracts were evaporated under reduced pressure to afford a crude extract, which was further dissolved in water and partitioned against EtOAc (10 L × 5) to yield 130 g of the extract. Five fractions (A-E) were eluted by gradient elution of chloroform/methanol (1:0-0:1, V/V) by normal silica gel column chromatography.

Fraction B (30 g) was eluted by MPLC (MeOH-H_2_O, 10:90-100:0, V/V) to obtain nine subfractions (B_1_–B_9_). Fraction B_3_ (380 mg) was separated by preparative HPLC using a reversed-phase column (CH_3_CN-H_2_O from 35:65 to 50:50 in 20 min, flow rate 4 mL/min) to yield **1** (20.3 mg, *t*_R_ = 15.6 min). Fraction B_4_ (6.7 g) was purified over Sephadex LH-20 eluted with MeOH to give six subfractions (B_4–1_–B_4–6_). Fraction B_4–3_ (198 mg) was further purified by preparative HPLC with CH_3_CN-H_2_O (from 32:68 to 52:48 in 20 min, flow rate 4 mL/min) to obtain Compounds **2** (30 mg, *t*_R_ = 16.8 min), **3** (7.8 mg, *t*_R_ = 17.2 min), **4** (6.6 mg, *t*_R_ = 28.3 min), and **5** (7.3 mg, *t*_R_ = 29.6 min). Fraction B_4–4_ (600 mg) was separated on a silica gel column and eluted with petroleum ether and ethyl acetate (10:1) to yield five subfractions (B_4–4–1_-B_4–4–5_). B_4–4–3_ was purified by prep-HPLC (CH_3_CN-H_2_O from 39:61 to 55:45 in 25 min, flow rate 4 mL/min) to give **6** (6.8 mg, *t*_R_ = 15.6 min), **7** (17.2 mg, *t*_R_ = 17.9 min) and **8** (20.4 mg, *t*_R_ = 20.8 min).

Fraction C (43 g) was eluted by MPLC (MeOH-H_2_O, 10:90-100:0, V/V) to obtain eleven subfractions (C_1_–C_11_). Fraction C_5_ (3.6 g) was separated on a silica gel column and eluted with petroleum ether and ethyl acetate (8:1), to yield eight subfractions (C_5–1_–C_5–8_). C_5–5_ was purified by prep-HPLC (CH_3_CN-H_2_O from 37:63 to 56:44 in 20 min, flow rate 4 mL/min) to give **9** (11.8 mg, *t*_R_ = 18.2 min) and **10** (9.5 mg, *t*_R_ = 19.7 min). Fraction C_6_ (12 g) was purified over Sephadex LH-20 eluted with MeOH to give nine subfractions (C_6–1_–C_6–9_). Fraction C_6–5_ (300 mg) was further purified by preparative HPLC with CH_3_CN-H_2_O (from 40:60 to 54:46 in 20 min, flow rate 4 mL/min) to obtain Compounds **11** (28 mg, *t*_R_ = 18.4 min), **12** (6.1 mg, *t*_R_ = 19 min) and **13** (10.4 mg, *t*_R_ = 23.6 min). Fraction C_7_ (380 mg) was separated by preparative HPLC using a reversed-phase column (CH_3_CN-H_2_O from 42:58 to 57:43 in 20 min, flow rate 4 mL/min) to yield **14** (16.3 mg, *t*_R_ = 18.5 min) and **15** (7.5 mg, *t*_R_ = 21.6 min).

### General experimental procedures

Both 1D and 2D spectra were run on Bruker Avance III 600 MHz and Bruker Avance III 500 MHz spectrometers. IR spectra were obtained with a Tenor 27 spectrophotometer using KBr pellets. UV spectra were measured on a UH-5300 spectrometer. CD spectra were recorded by a Chirascan-plus circular dichromatic spectrometer. The optical rotations were measured with a Horiba Sepa-300 polarimeter. According to the solvent signal, the chemical shifts were expressed in ppm. The mass spectra were recorded on a mass spectrometer (Thermo Fisher Scientific, Bremen, Germany). Medium-pressure liquid chromatography (MPLC) was applied to the Biotage SP1 system and packed with RP-18 gel. Preparative high-performance liquid chromatography (prep-HPLC) was performed on an Agilent 1260 liquid chromatography system equipped with Zorbax SB-C18 columns (5 μm, 9.4 mm × 150 mm, or 21.2 mm × 150 mm) and a DAD detector. Column chromatography (CC) separations were carried out in silica gel (200–300 mesh, Qingdao Haiyang Chemical Co., Ltd., Qingdao) and Sephadex LH-20 (Sweden’s France Asia Fine Chemical Co., Ltd.). The components were monitored by TLC (GF_254_, Qingdao Haiyang Chemical Co., Ltd., Qingdao) and the spots were observed by heating the silica gel plate and spraying with vanillin and 10% H_2_SO_4_ in ethanol.

### Spectroscopic characterization of Compounds 2–12

Paecilin F (**2**): light yellow crystal (MeOH); [α]^25.1^_D_ + 2.3 (*c*.5, MeOH); UV (MeOH) λ_max_ (log ε) 260 (3.28) nm; IR (KBr) ν_max_ 3500, 3000, 2950, 1794, 1736, 1647, 1458, and 1320 cm^–1^; ^1^H nuclear magnetic resonance (NMR) (500 MHz, CDCl_3_) and ^13^C NMR (125 MHz, CDCl_3_) data, see Table 1; positive ion HRESIMS *m/z* 693.1814 [M + Na]^+^ (calcd for C_33_H_34_O_15_Na, 693.1795).

**TABLE 1:** ^1^H and ^13^C NMR spectroscopic data of Compounds **1**–**3** (CDCl_3_).

No.	1*^a^*		2*^b^*		3*^b^*	
	δ_C_, type	δ_H_, mult (*J* in Hz)	δ_C_, type	δ_H_, mult (*J* in Hz)	δ_C_, type	δ_H_, mult (*J* in Hz)
2	84.4, C		86.0, C		87.0, C	
3	40.0, CH_2_	3.27, d (17.2) 3.15, d (17.2)	40.1, CH_2_	3.28, d (17.3) 3.16, d (17.3)	40.7, CH_2_	3.23, d (17.3) 3.19, d (17.3)
4	194.2, C		194.2, C		196.1, C	
4a	107.9, C		107.9, C		107.8, C	
5	161.9, C		161.9, C		161.8, C	
6	110.4, CH	6.62, d (8.6)	110.4, CH	6.63, d (8.6)	110.1, CH	6.60, d (8.6)
7	141.4, CH	7.45, d (8.6)	141.7, CH	7.48, d (8.6)	141.2, CH	7.47, d (8.6)
8	115.3, C		115.3, C		115.2, C	
8a	156.2, C		156.1, C		156.2, C	
9	82.9, CH	4.86, d (6.6)	82.4, CH	4.65, d (7.6)	76.6, CH	3.87, s
10	33.4, CH	2.95, m	34.1, CH	2.88, m	30.6, CH	2.27, m
11	37.0, CH_2_	2.73, dd (17.3, 8.2) 2.43, dd (17.3, 7.0)	35.4, CH_2_	2.28, dd (17.3, 8.4) 1.94, dd (17.3, 11.2)	40.0, CH_2_	2.46, dd (15.7, 6.6) 2.23, dd (15.7, 6.1)
12	175.0, C		174.7, C		173.3, C	
13	14.9, CH_3_	1.29, d (7.2)	14.7, CH_3_	1.23, d (7.2)	13.3, CH_3_	0.83, d (6.6)
14	169.0, C		168.8, C		170.6, C	
15	53.8, CH_3_	3.79, s	53.8, CH_3_	3.75, s	53.5, CH_3_	3.74, s
16					51.9, CH_3_	3.66, s
5-OH		11.58, s		11.60, s		11.69, s
2′	85.9, C		87.2, C		87.0, C	
3′	39.6, CH_2_	3.33, d (17.2) 3.20, d (17.2)	40.1, CH_2_	3.31, d (17.3) 3.24, d (17.3)	40.4, CH_2_	3.30, d (17.3) 3.25, d (17.3)
4′	194.7, C		196.5, C		196.0, C	
4a′	107.4, C		107.5, C		107.6, C	
5′	159.0, C		158.9, C		159.3, C	
6′	117.7, C		117.4, C		117.7, C	
7′	141.2, CH	7.58, d (8.6)	141.0, CH	7.62, d (8.6)	141.2, CH	7.68, d (8.6)
8′	107.9, CH	6.61, d (8.6)	108.0, CH	6.61, d (8.6)	107.3, CH	6.62, d (8.6)
8a′	158.4, C		158.9, C		159.0, C	
9′	82.4, CH	4.65, d (7.5)	76.3, CH	4.08, s	76.5, CH	4.06, s
10′	33.9, CH	2.87, m	30.9, CH	2.35, m	30.9, CH	2.38, m
11′	35.3, CH_2_	2.28, dd (17.3, 8.5) 1.93, dd (17.3, 11.1)	40.0, CH_2_	2.60, dd (18.0, 9.1) 2.38, m	40.1, CH_2_	2.61, dd (17.8, 9.0) 2.41, dd (17.8, 5.7)
12′	174.7, C		173.3, C		173.3, C	
13′	14.6, CH_3_	1.22, d (7.1)	13.9, CH_3_	1.06, d (6.6)	13.8, CH_3_	1.07, d (6.4)
14′	168.8, C		170.5, C		170.5, C	
15′	53.7, CH_3_	3.73, s	53.5, CH_3_	3.77, s	53.5, CH_3_	3.72, s
16′			51.9, CH_3_	3.70, s	51.9, CH_3_	3.70, s
5′-OH		11.82, s		11.95, s		11.96, s

^a^Measured on 600/150 MHz; ^b^Measured on 500/125 MHz.

Paecilin G (**3**): pale yellow gum; [α]^21.5^_D_ + 66.9 (*c*.5, MeOH); UV (MeOH) λ_max_ (log ε) 260 (2.70) nm; IR (KBr) ν_max_ 3500, 1734, 1647, 1472, 1437, 1356, 1281, 1233, 1198, 1177, 1070, 1049, and 1011 cm^–1^; ^1^H NMR (500 MHz, CDCl_3_) and ^13^C NMR (125 MHz, CDCl_3_) data, see Table 1; positive ion HRESIMS *m/z* 725.2048 [M + Na]^+^ (calcd for C_34_H_38_O_16_Na, 725.2058).

Paecilin H (**4**): pale yellow gum; [α]^21.4^_D_ + 69.6 (*c*.5, MeOH); UV (MeOH) λ_max_ (log ε) 260 (3.20) nm; IR (KBr) ν_max_ 3500, 1792, 1738, 1651, 1472, 1437, 1356, 1281, 1223, 1196, 1177, 1067, 1007, 837, 785, 584, and 536 cm^–1^; ^1^H NMR (500 MHz, CDCl_3_) and ^13^C NMR (125 MHz, CDCl_3_) data, see Table 2; positive ion HRESIMS *m/z* 693.1791 [M + Na]^+^ (calcd for C_33_H_34_O_15_Na 693.1795).

**TABLE 2:** ^1^H and ^13^C NMR spectroscopic data of Compounds **4**, **5,** and **7** (CDCl_3_).

No.	4*^a^*		5*^b^*		7*^b^*	
	δ_C_, type	δ_H_, mult (*J* in Hz)	δ_C_, type	δ_H_, mult (*J* in Hz)	δ_C_, type	δ_H_, mult (*J* in Hz)
2	84.7, C		85.0, C		85.7, C	
3	39.9, CH_2_	3.31, d (17.2) 3.19, d (17.2)	101.7, C		40.9, CH_2_	3.27, d (17.3) 3.19, d (17.3)
4	194.3, C		187.3, C		194.3, C	
4a	107.6, C		107.0, C		107.8, C	
5	159.3, C		159.4, C		161.9, C	
6	118.0, C		118.2, C		110.4, CH	6.63, d (8.7)
7	141.6, CH	7.72, d (8.6)	140.5, CH	7.66, d (8.6)	141.6, CH	7.76, d (8.7)
8	107.4, CH	6.63, d (8.6)	107.8, CH	6.64, d (8.6)	114.5, C	
8a	158.6, C		158.5, C		155.7, C	
9	82.8, CH	4.83, d (6.9)	77.2, CH	3.95, d (11.3)	82.6, CH	4.68, d (7.2)
10	33.6, CH	3.00, m	29.4, CH	2.44, m	33.8, CH	2.88, m
11	36.8, CH_2_	2.72, dd (17.3, 8.3) 2.50, dd (17.3, 8.0)	36.4, CH_2_	2.75, dd (19.3, 6.4) 2.33, dd (19.3, 10.6)	36.1, CH_2_	1.97, dd (17.2, 9.9) 2.42, dd (17.2, 8.3)
12	175.0, C		177.9, C		174.5, C	
13	15.0, CH_3_	1.35, d (7.2)	18.2, CH_3_	1.18, d (6.6)	14.8, CH_3_	1.18, d (7.2)
14	169.1, C		170.4, C		168.8, C	
15	53.5, CH_3_	3.76, s	53.5, CH_3_	3.73, s	53.9, CH_3_	3.79, s
16						
5-OH		11.86, s		11.67, s		11.70, s
9-OH				2.83, s		
12-OH				13.76, s		
2′	87.0, C		87.0, C		87.0, C	
3′	40.8, CH_2_	3.24, d (17.2) 3.20, d (17.2)	40.5, CH_2_	3.24, d (17.5) 3.20, d (17.5)	40.0, CH_2_	3.27, d (17.3) 3.19, d (17.3)
4′	196.0, C		196.0, C		195.8, C	
4a′	107.8, C		107.8, C		107.7, C	
5′	161.9, C		161.8, C		161.8, C	
6′	110.1, CH	6.61, d (8.6)	110.2, CH	6.61, d (8.6)	110.9, CH	6.65, d (8.7)
7′	141.1, CH	7.47, d (8.6)	141.2, CH	7.47, d (8.6)	142.2, CH	7.92, d (8.7)
8′	115.0, C		115.4, C		114.9, C	
8a′	156.2, C		156.1, C		155.8, C	
9′	76.7, CH	3.88, d (2.0)	76.6, CH	3.88, s	76.8, CH	3.89, dd (7.9, 1.6)
10′	30.6, CH	2.29, m	30.8, CH	2.26, m	30.8, CH	2.28, m
11′	40.0, CH_2_	2.46, dd (15.7, 6.5) 2.23, dd (15.7, 6.0)	39.9, CH_2_	2.46, dd (15.6, 6.5) 2.22, dd (15.6, 6.3)	39.9, CH_2_	2.22, dd (15.6, 6.3) 2.46, dd (15.6, 6.3)
12′	173.3, C		173.2, C		173.2, C	
13′	13.9, CH_3_	0.84, d (6.7)	13.6, CH_3_	0.87, d (6.6)	13.3, CH_3_	0.83, d (6.5)
14′	170.5, C		170.4, C		170.5, C	
15′	53.8, CH_3_	3.72, s	53.4, CH_3_	3.73, s	53.6, CH_3_	3.78, s
16′	51.9, CH_3_	3.66, s	51.9, CH_3_	3.65, s	51.9, CH_3_	3.65, s
5′-OH		11.70, s		11.70, s		11.68, s
9′-OH				2.55, s		2.50, d (7.9)

^a^Measured on 600/150 MHz; ^b^Measured on 500/125 MHz.

Paecilin I (**5**): pale yellow gum; [α]^21.4^_D_ + 147.3 (*c*.5, MeOH); UV (MeOH) λ_max_ (log ε) 265 (2.39) nm; IR (KBr) ν_max_ 3500, 1734, 1653, 1616, 1458, 1356, 1273, 1233, 1067, 1049, 1016, and 820 cm^–1^; ^1^H NMR (600 MHz, CDCl_3_) and ^13^C NMR (150 MHz, CDCl_3_) data, see Table 2; positive ion HRESIMS *m/z* 693.1810 [M + Na]^+^ (calcd for C_33_H_34_O_15_Na, 693.1795).

Paecilin J (**6**): brown crystal (MeOH); [α]^22.5^_D_ + 36.9 (*c*.5, MeOH); UV (MeOH) λ_max_ (log ε) 260 (1.40) nm; IR (KBr) ν_max_ 3500, 2922, 2851, 1790, 1742, 1647, 1466, 1383, 1346, 1234, 1202, 1179, 1049, and 1024 cm^–1^; ^1^H NMR (500 MHz, CD_3_COCD_3_) and ^13^C NMR (125 MHz, CD_3_COCD_3_) data, see Table 3; positive ion HRESIMS *m/z* 661.1526 [M + Na]^+^ (calcd for C_32_H_30_O_14_Na 661.1533).

**TABLE 3:** ^1^H and ^13^C NMR spectroscopic data of Compounds **6**, **8,** and **12** (CDCl_3_).

No.	6^*ac*^		8^*ad*^		12^*bd*^	
	δ_C_, type	δ_H_, mult (*J* in Hz)	δ_C_, type	δ_H_, mult (*J* in Hz)	δ_C_, type	δ_H_, mult (*J* in Hz)
2,2′	86.9, C		84.6, C		86.9, C	
3,3′	40.4, CH_2_	3.56, d (17.6) 3.18, d (17.6)	39.8, CH_2_	3.27, d (17.3) 3.19, d (17.3)	40.3, CH_2_	3.26, d (17.3) 3.23, d (17.3)
4,4′	195.9, C		194.2, C		196.1, C	
4a,4a′	108.4, C		107.6, C		107.6, C	
5,5′	162.3, C		159.2, C		159.3, C	
6,6′	110.8, CH	6.54, d (8.7)	117.7, C		117.6, C	
7,7′	142.5, CH	7.89, d (8.7)	141.3, CH	7.52, d (8.6)	141.0, CH	7.50, d (8.5)
8,8′	115.4, C		107.5, CH	6.62, d (8.6)	107.3, CH	6.61, d (8.5)
8a,8a′	156.7, C		158.6, C		158.9, C	
9,9′	82.9, CH	4.89, d (7.2)	82.7, CH	4.80, d (6.8)	76.5, CH	4.05, s
10,10′	34.4, CH	3.03, m	33.6, CH	2.98, m	30.9, CH	2.37, m
11,11′	36.6, CH_2_	2.39, dd (17.0, 8.3) 1.85, dd (17.0, 9.9)	36.8, CH_2_	2.70, dd (17.3, 8.3) 2.47, dd (17.3, 8.3)	40.1, CH_2_	2.60, dd (17.9, 9.1) 2.40, dd (17.9, 9.1)
12,12′	175.1, C		175.0, C		173.3, C	
13,13′	15.0, CH_3_	1.20, d (7.2)	15.0, CH_3_	1.33, d (7.2)	13.8, CH_3_	1.06, d (6.5)
14,14′	170.0, C		169.1, C		170.6, C	
15,15′	53.9, CH_3_	3.80, s	53.8, CH_3_	3.76, s	53.6, CH_3_	3.76, s
16,16′					51.9, CH_3_	3.70, s
5/5′-OH		11.70, brs		11.91, s		12.00, s
9/9′-OH						2.77, s

^a^Measured on 500/125 MHz; ^b^Measured on 600/150 MHz; ^c^Measured in CD_3_COCD_3_; ^d^Measured in CDCl_3_.

Paecilin K (**7**): pale yellow gum; [α]^21.3^_D_ + 46 (*c*.5, MeOH); UV (MeOH) λ_max_ (log ε) 260 (1.76) nm; IR (KBr) ν_max_ 3500, 1794, 1740, 1647, 1468, 1348, 1283, 1236, 1051, 1020, 837, 783, 758, 737, 648, and 550 cm^–1^; ^1^H NMR (500 MHz, CDCl_3_) and ^13^C NMR (125 MHz, CDCl_3_) data, see Table 2; positive ion HRESIMS *m/z* 693.1785 [M + Na]^+^ (calcd for C_33_H_34_O_15_Na, 693.1795).

Paecilin L (**8**): pale yellow gum; [α]^21.0^_D_ + 52.9 (*c*.5, MeOH); UV (MeOH) λ_max_ (log ε) 265 (1.85) nm; IR (KBr) ν_max_ 3500, 2920, 1792, 1740, 1645, 1626, 1435, 1362, 1215, 1177, 1150, 1061, 1024, 804, and 586 cm^–1^; ^1^H NMR (500 MHz, CDCl_3_) and ^13^C NMR (125 MHz, CDCl_3_) data, see Table 3; positive ion HRESIMS m/z 661.1538 [M + Na]^+^ (calcd for C_32_H_30_O_14_Na, 661.1533).

Paecilin M (**9**): pale yellow gum; [α]^20.8^_D_ – 96.9 (*c*.5, MeOH); UV (MeOH) λ_max_ (log ε) 265 (1.66) nm; IR (KBr) ν_max_ 3500, 2924, 1788, 1736, 1626, 1578, 1437, 1362, 1288, 1271, 1250, 1217, 1119, 1065, 839, 816, 785, and 586 cm^–1^; ^1^H NMR (600 MHz, CDCl_3_) and ^13^C NMR (150 MHz, CDCl_3_) data, see Table 4; positive ion HRESIMS *m/z* 679.1627 [M + Na]^+^ (calcd for C_32_H_32_O_15_Na, 679.1639).

**TABLE 4:** ^1^H and ^13^C NMR spectroscopic data of Compounds **9**, **10,** and **11** (CDCl_3_).

No.	9*^a^*		10*^b^*		11*^a^*	
	δ_C_, type	δ_H_, mult (*J* in Hz)	δ_C_, type	δ_H_, mult (*J* in Hz)	δ_C_, type	δ_H_, mult (*J* in Hz)
2	84.5, C		84.5, C		84.6, C	
3	39.9,CH_2_	3.26, d (17.3) 3.23, d (17.3)	39.7, CH_2_	3.26, d (17.3) 3.22, d (17.3)	39.9, CH_2_	3.27, d (17.2) 3.20, d (17.2)
4	196.1, C		196.2, C		194.2, C	
4a	107.7, C		107.6, C		107.7, C	
5	159.3, C		159.2, C		159.3, C	
6	117.5, C		117.3, C		117.4, C	
7	141.0, CH	7.49, d (8.6)	140.9, CH	7.47, d (8.6)	141.4, CH	7.53, d (8.5)
8	107.5, CH	6.62, d (8.6)	107.4, CH	6.61, d (8.6)	107.5, CH	6.63, d (8.5)
8a	159.0, C		159.0, C		158.5, C	
9	82.8, CH	4.80, d (6.9)	82.7, CH	4.79, d (6.9)	82.8, CH	4.80, d (6.9)
10	33.6, CH	2.98, m	33.6, CH	2.99, m	33.7, CH	2.98, m
11	36.8, CH_2_	2.70, dd (17.3, 8.3) 2.49, dd (17.3, 8.1)	36.7, CH_2_	2.69, dd (17.3, 8.3) 2.47, dd (17.3, 8.1)	36.8, CH_2_	2.70, dd (17.3, 8.3) 2.49, dd (17.3, 8.1)
12	175.1, C		175.2, C		175.0, C	
13	15.0, CH_3_	1.33, d (7.2)	14.9, CH_3_	1.32, d (7.2)	15.0, CH_3_	1.34, d (7.2)
14	169.2, C		169.2, C		169.2, C	
15	53.9, CH_3_	3.75, s	53.8, CH_3_	3.75, s	53.9, CH_3_	3.77, s
**16**						
5-OH		12.00, s		12.00, s		12.00, s
2′	86.9, C		87.0, C		87.0, C	
3′	40.1, CH_2_	3.24, d (17.3) 3.19, d (17.3)	40.2, CH_2_	3.25, d (17.3) 3.18, d (17.3)	40.3, CH_2_	3.27, d (17.5) 3.23, d (17.5)
4′	194.3, C		194.2, C		196.1, C	
4a′	107.6, C		107.5, C		107.7, C	
5′	159.3, C		159.2, C		159.3, C	
6′	117.8, C		117.8, C		117.9, C	
7′	141.4, CH	7.52, d (8.6)	141.3, CH	7.51, d (8.6)	141.0, CH	7.50, d (8.5)
8′	107.4, CH	6.60, d (8.6)	107.3, CH	6.58, d (8.6)	107.4, CH	6.62, d (8.5)
8a′	158.5, C		158.5, C		159.1, C	
9′	76.3, CH	4.08, s	76.3, CH	4.03, d (2.0)	76.5, CH	4.06, dd (5.9, 1.5)
10′	30.8, CH	2.39, m	30.9, CH	2.35, m	30.9, CH	2.39, m
11′	39.9, CH_2_	2.65, dd (16.9, 7.0) 2.42, dd (16.9, 5.3)	40.0, CH_2_	2.58, dd (17.9, 9.2) 2.38, dd (17.9, 5.9)	40.4, CH_2_	2.58, dd (17.7, 9.1) 2.36, dd (17.7, 5.8)
12′	173.3, C		173.3, C		172.9, C	
13′	13.8, CH_3_	1.09, d (6.6)	13.8, CH_3_	1.05, d (6.4)	13.7, CH_3_	1.07, d (6.6)
14′	170.6, C		170.6, C		170.6, C	
15′	53.6, CH_3_	3.74, s	53.5, CH_3_	3.74, s	53.6, CH_3_	3.76, s
16′			51.9, CH_3_	3.68, s	60.9, CH_2_	4.16, q (7.1)
17′					14.4, CH_3_	1.28, t (7.1)
5′-OH		11.89, s		11.87, s		11.87, s

^a^Measured on 600/150 MHz; ^b^Measured on 500/125 MHz.

Paecilin N (**10**): pale yellow gum; [α]^25.1^_D_ – 62.7 (*c*.5, MeOH); UV (MeOH) λ_max_ (log ε) 260 (1.88) nm; IR (KBr) ν_max_ 3500, 2960, 1800, 1736, 1624, 1540, 1437, 1320, 1063, and 800 cm^–1^; ^1^H NMR (500 MHz, CDCl_3_) and ^13^C NMR (125 MHz, CDCl_3_) data, see Table 4; positive ion HRESIMS *m/z* 671.2001 [M + H]^+^ (calcd for C_33_H_35_O_15_, 671.1976).

Paecilin O (**11**): pale yellow gum; [α]^25.1^_D_ – 46.7 (*c*.5, MeOH); UV (MeOH) λ_max_ (log ε) 260 (1.37) nm; IR (KBr) ν_max_ 3500, 2970, 1792, 1734, 1626, 1520, 1480, and 1204 cm^–1^; ^1^H NMR (600 MHz, CDCl_3_) and ^13^C NMR (150 MHz, CDCl_3_) data, see Table 4; positive ion HRESIMS *m/z* 707.1934 [M + Na]^+^ (calcd for C_34_H_36_O_15_Na, 707.1952).

Paecilin P (**12**): pale yellow gum; [α]^21.4^_D_ – 59.6 (*c*.5, MeOH); UV (MeOH) λ_max_ (log ε) 265 (3.25) nm; IR (KBr) ν_max_ 3500, 1736, 1647, 1626, 1437, 1364, 1204, 1119, 1065, 1011, and 584 cm^–1^; ^1^H NMR (600 MHz, CDCl_3_) and ^13^C NMR (150 MHz, CDCl_3_) data, see Table 3; positive ion HRESIMS *m/z* 725.2049 [M + Na]^+^ (calcd for C_34_H_38_O_16_Na, 725.2058).

### X-ray crystallographic analysis of Compounds 1, 2 and 6

#### X-ray crystallographic analysis of paecilin A (1)

C_32_H_30_O_14_, *M* = 638.56, *a* = 13.492(2) Å, *b* = 8.2053(15) Å, *c* = 14.726(3) Å, α = 90°, β = 114.945(5)°, γ = 90°, *V* = 1488.7(5) Å^3^, *T* = 294(2) K, space group P1211, *Z* = 2, μ(Cu Kα) = 1.54178 mm^–1^. The final anisotropic full-matrix least-squares refinement on F^2^ with 424 variables converged at R_1_ = 2.99% for the observed data and wR^2^ = 8.38% for all data. The goodness-of-fit was 1.061. The absolute configuration was determined by the Flack parameter = 0.03(5). CCDC: 2155089. Available online: https://www.ccdc.cam.ac.uk (accessed on 28 February 2022).

#### X-ray crystallographic analysis of paecilin F (2)

C_33_H_34_O_15_, *M* = 670.6, *a* = 14.3975(11) Å, *b* = 14.4598(11) Å, *c* = 15.2705(12) Å, α = 90°, β = 90°, γ = 90°, *V* = 3179.1(4) Å^3^, *T* = 295(2). K, space group P212121, *Z* = 4, μ(Cu Kα) = 1.54178 mm^–1^. The final anisotropic full-matrix least-squares refinement on F^2^ with 448 variables converged at R_1_ = 3.63% for the observed data and wR^2^ = 10.01% for all data. The goodness-of-fit was 1.058. The absolute configuration was determined by the Flack parameter = 0 (3). CCDC: 2155090. Available online: https://www.ccdc.cam.ac.uk (accessed on 28 February 2022).

#### X-ray crystallographic analysis of paecilin J (6)

C_32_H_30_O_14_, *M* = 638.56, *a* = 12.0791(14) Å, *b* = 13.3887(17) Å, *c* = 18.223(2) Å, α = 90°, β = 126.144(2)°, γ = 90°, *V* = 2947.1(6) Å^3^, *T* = 299(2) K, space group C2221, *Z* = 4, μ(Cu Kα) = 1.54178 mm^–1^. The final anisotropic full-matrix least-squares refinement on F^2^ with 212 variables converged at R_1_ = 3.67% for the observed data and wR^2^ = 11.05% for all data. The goodness-of-fit was 1.18. The absolute configuration was determined by the Flack parameter = 0.04(8). CCDC: 2155091. Available online: https://www.ccdc.cam.ac.uk (accessed on 28 February 2022).

### Equivalent circulating density calculations

Equivalent circulating density (ECD) calculations were carried out by the Gaussian 16 software package (Frisch et al., 2013). The conformation of the system was analyzed by Spartan 14 and calculated by the MMFF94s molecular mechanics’ force field. The cutoff energy was 10 kcal/mol. The conformational optimization and frequency were calculated at the B3LYP/def2svp theoretical level by using the IEFPCM solvent model (MeOH). The optimized conformations with proportions greater than 2% were selected for ECD calculations. Compounds **3**, **4**, **8**, **10**, **11**, and **12** used the IEFPCM solvent model (MeOH) to calculate ECD (TDDFT) at the wB97xd/def2svp theoretical level. Compounds **5** and **9** used the IEFPCM solvent model (MeOH) to calculate ECD (TDDFT) at the B3LYP/DGD2VP theoretical level. Compound **7** used the IEFPCM solvent model (MeOH) to calculate ECD (TDDFT) at the wB97xd/TZVP theoretical level. The ECD curves were simulated in SpecDis v1.71 using a Gaussian function (Bruhn et al., 2013). The calculated ECD data of all conformers were Boltzmann averaged by Gibbs free energies.

### Antimicrobial activity assays

The Microbroth dilution drug sensitivity test was used for MIC determination of the tested compounds. The bacterial liquid growth was observed on a clean workbench when the concentrations of the compounds to be measured were 8, 16, 32, 64, 128, 256, and 512 μg/mL. The concentrations of *Escherichia coli* ATCC 25922 and *Salmonella enteritidis* ATCC 25923 after secondary activation were adjusted to 1.0 × 10^8^ CFU/mL using MH medium (Qingdao High-tech Industrial Park Haibo Biotechnology Co., Ltd.). The concentration of *C. albicans* ATCC 10231 was adjusted to 1.0 × 10^5^ CFU/mL using a PDB medium (Qingdao High-tech Industrial Park Haibo Biotechnology Co., Ltd.). The 96-well plate was rationally planned, and the experimental groups were set as 100 μL compound and 100 μL bacterial solution, with two multiple Wells. The first group of negative control medium was 200 μL MH or PDB medium, which was evaluated in a single well. In the second group, 100 μL DMSO and 100 MH or PDB medium were used as negative controls. The positive control was a 200 μL bacterial solution with two duplicate wells. Amphenicol, penicillin, and fluconazole were used as positive controls for *E. coli*, *S. enteritidis*, and *C. albicans*, respectively. The 96-well plate was cultured in a high-throughput growth curve analyzer for 24 h, and the results were observed. The positive control well showed turbidity, while the negative control was clear. The quality control was within the specified range, and the MIC values of compounds were the lowest concentrations without bacterial growth observed by the naked eye.

## Results and discussion

### Structure elucidations

**FIGURE 2:**
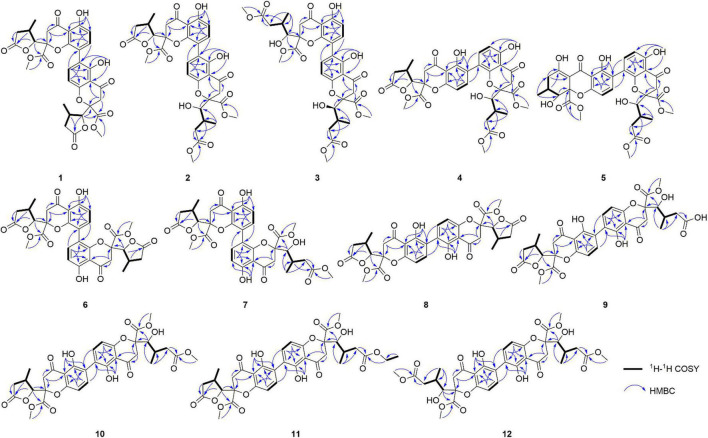
Key ^1^H-^1^H COSY and HMBC correlations for **1**–**12**.

Compound **1** was obtained as light-yellow crystals, whose molecular formula was determined to be C_32_H_30_O_14_ by HRESIMS at *m/z* 661.1532 [M + Na]^+^ (calcd for C_32_H_30_O_14_Na^+^, 661.1533), with 18 degrees of unsaturation. In the ^1^H NMR spectrum (Table 1), two hydroxy protons at δ_*H*_ 11.58 (1H, s, 5-OH) and 11.82 (1H, s, 5′-OH), two pairs of adjacent aromatic protons at δ_*H*_ 7.58 (1H, d, *J* = 8.6 Hz, H-7′), 6.61 (1H, d, *J* = 8.6 Hz, H-8′), 7.45 (1H, d, *J* = 8.6 Hz, H-7) and 6.62 (1H, d, *J* = 8.6 Hz, H-6), two methoxy protons at δ_H_ 3.79 (3H, s, H-15) and 3.73 (3H, s, H-15′), two methyl doublets at δ_H_ 1.29 (3H, d, *J* = 7.2 Hz, H-13) and 1.22 (3H, d, *J* = 7.1 Hz, H-13′), two oxymethine protons at δ_H_ [4.86 (1H, d, *J* = 6.6 Hz, H-9) and 4.65 (1H, d, *J* = 7.5 Hz, H-9′) were clearly shown. The ^13^C NMR and DEPT spectra (Table 1) of **1** showed oxymethine carbons, including four methyl carbons (two oxygenated carbons), four methylene carbons, eight methine carbons (four sp^2^ carbons), and 16 quaternary carbons (two ketone carbonyl carbons, four ester carbonyl carbons, and eight sp^2^ carbons). The ^1^H NMR and ^13^C NMR spectral data (Table 1) of Compound **1** were nearly the same as those of paecilin A (Guo et al., 2007). Paecilin A is a symmetric dimer and should have one set of NMR data; however, there are two sets of data in its nuclear magnetic attribution table in the reference (Guo et al., 2007), so the structure of paecilin A was incorrectly assigned by erroneously linking C-8 with C-8′. Actually, C-8 was linked with C-6′, which was confirmed by the HMBC correlations from 5′-OH (δ_H_ 11.82) to δ_C_ C-6′ (δ_C_ 117.7)/C-4a’ (δ_C_ 107.4), from H-7 (δ_H_ 7.45) to C-6′ (δ_C_ 117.7), and from H-7′ (δ_H_ 7.58) to C-8 (δ_C_ 115.3) (Figure 2). The *cis* configurations of H-9/H-10 and H-9′/H-10′ in the γ-butyrolactone moiety were supported by the coupling constants (^3^*J*_H–9,H–10_ = 6.6 Hz, ^3^*J*_H–9’,H–10’_ = 7.5 Hz) (Wu et al., 2015). In addition, the relative configuration of Compound **1** was deduced as 2S*,9R*,10S*,2′S*,9′R*,10′S* by comparing the chemical shifts of H-2′/H-3′/H-4′ and the coupling constants of the four differential isomers in the reference (Tietze et al., 2014). Finally, the structure of **1** was confirmed by single-crystal X-ray diffraction analysis [Flack parameter = 0.03(5), CCDC: 2155089; Figure 3]. The absolute configuration of **1** was determined to be 2*S*,9*R*,10*S*,2′*S*,9′*R*,10′*S*. Therefore, the structure of paecilin A was revised.

**FIGURE 3:**
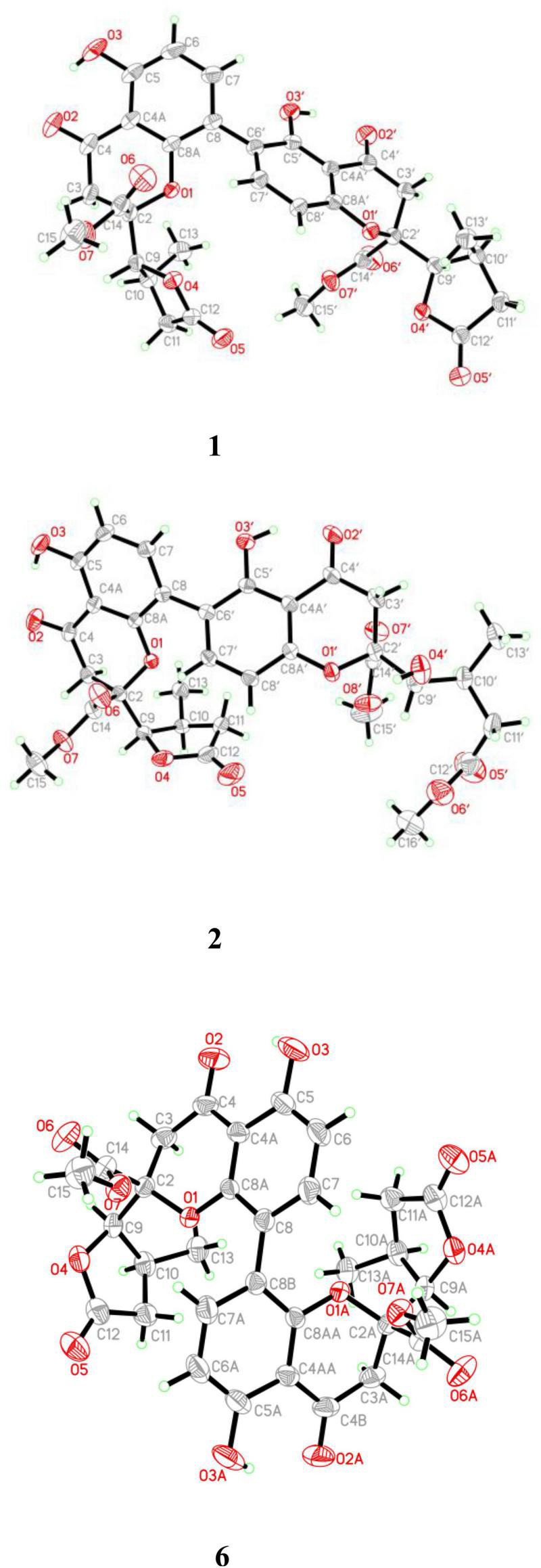
ORTEP drawing of Compounds **1**, **2,** and **6**.

Compound **2** was obtained as light-yellow crystals, whose molecular formula was determined to be C_33_H_34_O_15_ by HRESIMS analysis at *m/z* 693.1814 [M + Na]^+^ (calcd for C_33_H_34_O_15_Na^+^, 693.1795), with 17 degrees of unsaturation. Analysis of the ^1^H and ^13^C NMR data (Table 1) suggested that the structure of **2** was similar to that of **1**, and the only observed difference was that **2** has an additional methoxy group compared to that of **1.** Combined with the unsaturation degree, the γ-butyrolactone ring of one monomer part was opened. This change was confirmed by the key HMBC correlations (Figure 2) from H_3_-16′ (δ_H_ 3.70) to C-12′ (δ_C_ 173.3). By comparing the chemical shifts and coupling constants of H-9′ of Compound **2** (δ_H_ 4.08, s) with H-5′ in versixanthones E and F (δ_H_ 4.06, d, *J* = 1.8 Hz) (Wu et al., 2015), the relative configuration of Compound **2** was thus determined to be 2S*,9R*,10S*,2′S*,9′R*,10′S*. Finally, the absolute configuration of Compound **2** was determined to be 2*S*,9*R*,10*S*,2′*S*,9′*R*,10′*S* by single-crystal X-ray diffraction [Flack parameter = 0.00(3), CCDC: 2155090; Figure 3]. In conclusion, Compound **2** was named paecilin F.

**FIGURE 4:**
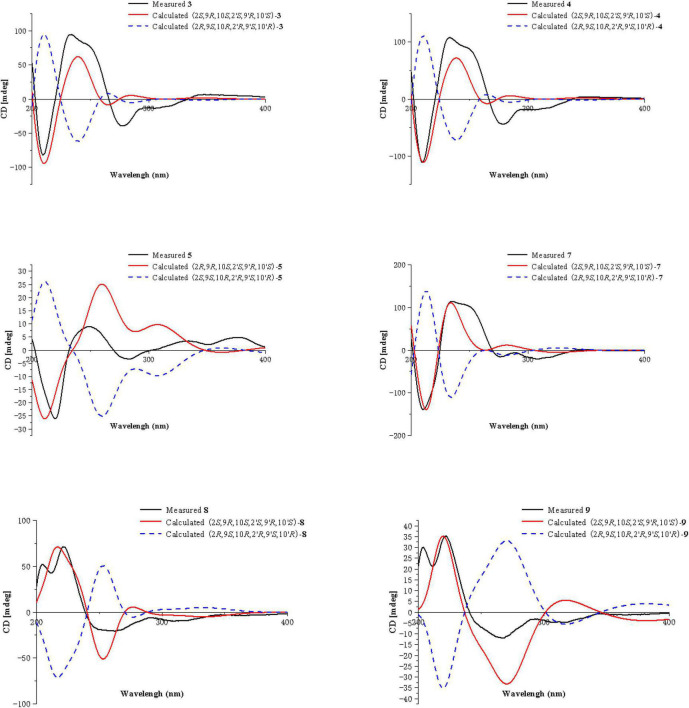
ECD calculations of **3**, **4**, **5**, **7**, **8,** and **9**.

Compound **3** was obtained as a pale-yellow gum, whose molecular formula was determined to be C_34_H_38_O_16_ by HRESIMS analysis at *m/z* 725.2048 [M + Na]^+^ (calcd for C_34_H_38_O_16_Na^+^, 725.2058), with 16 degrees of unsaturation. The ^1^H and ^13^C NMR spectroscopic data of **3** (Table 1) showed that **3** was a congener of **2**. The only difference was that Compound **3** has an additional methoxy group compared to **2**. By considering the degrees of unsaturation, it was suggested that the γ-butyrolactone groups of the two monomer moieties were opened. These changes were confirmed by the key HMBC correlations (Figure 2) from H_3_-16 (δ_H_ 3.66) to C-12 (δ_C_ 173.3) and H_3_-16′ (δ_H_ 3.70) to C-12′ (δ_C_ 173.3). Referring to the chemical shifts and coupling constants of **2**, the relative configuration of Compound **3** was deduced as 2S*,9R*,10S*,2′S*,9′R*,10′S*. Finally, the absolute configuration of Compound **3** was determined to be 2*S*,9*R*,10*S*,2′*S*,9′*R*,10′*S* by ECD calculations (Figure 4). In summary, the structure of **3** was established and named paecilin G.

Compound **4** was obtained as a pale-yellow gum. Its molecular formula of C_33_H_34_O_15_ was determined based on the HRESIMS data (found at *m/z* 693.1791 [M + Na]^+^, calcd for C_33_H_34_O_15_Na^+^ 693.1795), corresponding to 17 degrees of unsaturation. Compound **4** had the same molecular formula and highly similar 1D NMR data (Table 2) to those of **2**. The only difference was that the two monomers of Compound **4** were connected by C-6 and C-8′. The ^1^H-^1^H COSY cross peaks (Figure 2) of H-7 (δ_H_ 7.72)/H-8 (δ_H_ 6.63), H-6′ (δ_H_ 6.61)/H-7′ (δ_H_ 7.47), and the key HMBC correlations from H-7′ (δ_H_ 7.47) to C-6 (δ_C_ 118), from H-7 (δ_H_ 7.72) to C-8′ (δ_C_ 115), from 5-OH (δ_H_ 11.86) to C-4a (δ_C_ 107.6)/C-5 (δ_C_ 159.3)/C-6 (δ_C_ 118), and from 5′-OH (δ_H_ 11.7) to C-4a’ (δ_C_ 107.8)/C-5′ (δ_C_ 161.9)/C-6′ (δ_C_ 110.1) confirmed the above inference. The relative configuration of Compound **4** was deduced by comparison with the chemical shifts and coupling constants of **2**. Finally, the absolute configuration of Compound **4** was determined to be 2*S*,9*R*,10*S*,2′*S*,9′*R*,10′*S* by ECD calculations (Figure 4). In summary, the structure of **4** was established and named paecilin H.

**FIGURE 5:**
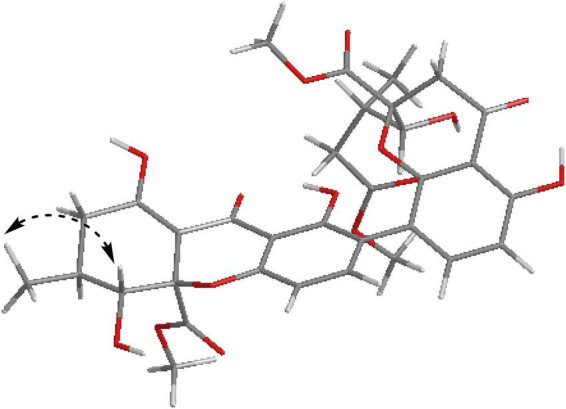
Key ROESY correlations for **5**.

Compound **5** was obtained as a pale-yellow gum, and it possessed the molecular formula of C_33_H_34_O_15_ as indicated by the HRESIMS analysis at *m/z* 693.1810 [M + Na]^+^ (calcd for C_33_H_34_O_15_Na^+^, 693.1795), with 17 degrees of unsaturation. The ^1^H and ^13^C NMR spectroscopic data of Compound **5** (Table 2) were highly similar to those of versixanthone B (Wu et al., 2015). The mass spectrometry data showed that Compound **5** had one more methoxy group than versixanthone B, which suggested that Compound **5** was the γ-butyrolactone ring-opening analog of versixanthone B. This conclusion was confirmed by the correlation between H-11′ (δ_H_ 2.46, 2.22) and H-16′ (δ_H_ 3.65 to C-12′) (δ_C_ 173.2) in the HMBC spectrum (Figure 2). Therefore, the planar structure of **5** was determined. The relative configuration of the tetrahydroxanthone monomer was readily established to be the same as that of versixanthone A by the coupling constant (^3^*J*_H–9,H–10_ = 11.3 Hz), the NOESY correlation between H-9 and H_3_-13 (Figure 5), and the chemical shifts. By considering the biosynthesis of this family of congeners, together with the chemical shifts, and coupling constants of H-5′, H-6′, and H-7′ in versixanthone E (Wu et al., 2015), it was deduced that the relative configuration of Compound **5** was 2R*,9R*,10S*,2′S*,9′R*,10′S*. Finally, the absolute configuration was deduced as 2*R*,9*R*,10*S*,2′*S*,9′*R*,10′*S* by ECD calculations (Figure 4). Therefore, Compound **5** could be fully assigned and named paecilin I.

Compound **6** was obtained as a brown crystal, whose molecular formula was determined to be C_32_H_30_O_14_ by HRESIMS analysis at *m/z* 661.1526 [M + Na]^+^ (calcd for C_32_H_30_O_14_Na^+^, 661.1533), with 18 degrees of unsaturation. According to the ^1^H NMR and ^13^C NMR data (Table 3) and the mass spectra, it could be inferred that Compound **6** was also a dimeric compound that was structurally similar to Compound **1**. There was only one set of ^1^H and ^13^C NMR spectroscopic data for **6**, indicating that the structure of **6** was symmetric. Therefore, Compound **6** had either a C8-C8′ or a C6-C6′ linkage pattern. Due to the lack of the key HMBC correlations of the signals 5-OH and 5′-OH, the connection position of its monomer remained to be determined. Fortunately, single-crystals of **6** were obtained, which finally enabled us to determine the absolute configuration as 2*S*,9*R*,10*S*,2′*S*,9′*R*,10′*S* [Flack parameter = 0.04(8), CCDC: 2155091; Figure 3]. In summary, the structure of **6** was established and named paecilin J.

Compound **7** was obtained as a pale-yellow gum, whose molecular formula was determined to be C_33_H_34_O_15_ by HRESIMS analysis at *m/z* 693.1785 [M + Na]^+^ (calcd for C_33_H_34_O_15_Na^+^, 693.1795), with 17 degrees of unsaturation. By comparing the ^1^H and ^13^C NMR spectra of Compound **7** (Table 2) with those of **2** (Table 1), it was found that **7** was a structural congener of **2**. The only difference between the two compounds was that Compound **7** was linked *via* C8-C8′ while **2** was linked by C8-C6′. This change was proved by the key HMBC correlations (Figure 2) from H-7′ (δ_H_ 7.92) to C-8 (δ_C_ 114.5), from H-7 (δ_H_ 7.76) to C-8′ (δ_C_ 114.9), from 5-OH (δ_H_ 11.70) to C-4a (δ_C_ 107.8)/C-5 (δ_C_ 161.9)/C-6 (δ_C_ 110.4), and from 5′-OH (δ_H_ 11.68) to C-4a′ (δ_C_ 107.7)/C-5′ (δ_C_ 161.8)/C-6′ (δ_C_ 110.9). By comparison of the coupling constants of Compounds **7** and **2**, it was deduced that the relative configuration of Compound **7** was 2S*,9R*,10S*,2′S*,9′R*,10′S*. Finally, the absolute configuration of Compound **7** was determined to be 2*S*,9*R*,10*S*,2′*S*,9′*R*,10′*S* by ECD calculations (Figure 4). Therefore, Compound **7** was elucidated and named paecilin K.

Compound **8** was obtained as a pale-yellow gum, whose molecular formula was determined to be C_32_H_30_O_14_ by the HRESIMS analysis at *m/z* 661.1538 [M + Na]^+^ (calcd for C_32_H_30_O_14_Na^+^, 661.1533), with 18 degrees of unsaturation. The 1D NMR spectrum of Compound **8** (Table 3) showed high similarity to that of Compound **6** (Table 3). The only difference was that the two monomers of Compound **6** were C8-C8′ linked, while the two monomers of Compound **8** were C6-C6′ linked. This conclusion was established by the key HMBC correlations (Figure 2) from H-7/H-7′ (δ_H_ 7.52) to C-6/C-6′ (δ_C_ 117.7), from 5-OH/5′-OH (δ_H_ 11.91) to C-4a/C-4a’ (δ_C_ 107.6), C-5/C-5′ (δ_C_ 159.2), and C-6/C-6′ (δ_C_ 117.7). By biosynthetic considerations, and comparison with the chemical shifts and coupling constants with those of **6**, the relative configuration of Compound **8** was deduced as 2S*,9R*,10S*,2′S*,9′R*,10′R*. The ECD calculation for **8** was performed, and the results of **8** matched well with the experimental ECD curve (Figure 4). Therefore, the absolute configuration of **8** was 2*S*,9*R*,10*S*,2′*S*,9′*R*,10′*S*, and it was named paecilin L.

Compound **9** was obtained as a pale-yellow gum, whose molecular formula was determined to be C_32_H_32_O_15_ by HRESIMS analysis at *m/z* 679.1627 [M + Na]^+^ (calcd for C_32_H_32_O_15_Na^+^, 679.1639), with 17 degrees of unsaturation. Comparing the ^1^H and ^13^C NMR data of Compounds **8** (Table 3) and **9** (Table 4), it was found that these two compounds are structural analogs. The mass spectrum showed that Compound **9** was 18 Da more than Compound **8**, corresponding to a molecule of H_2_O. Combined with the degrees of unsaturation, it was speculated that one of the γ-butyrolactone rings was opened. In the HMBC spectrum (Figure 2), H-9′ (δ_H_ 4.80) of Compound **8** was correlated with C-12′ (δ_C_ 175.0), while for Compound **9**, this correlation was absent. This evidence helped to prove the above conclusion. The relative configurations in the two monomeric units of **9** were proposed to be the same as **8**, as indicated by the similar chemical shifts and coupling constants and by consideration of the biogenetic origin. Finally, the absolute configuration of Compound **9** was determined to be 2*S*,9*R*,10*S*,2′*S*,9′*R*,10′*S* by ECD calculations (Figure 2). In summary, Compound **9** was named paecilin M.

**FIGURE 6:**
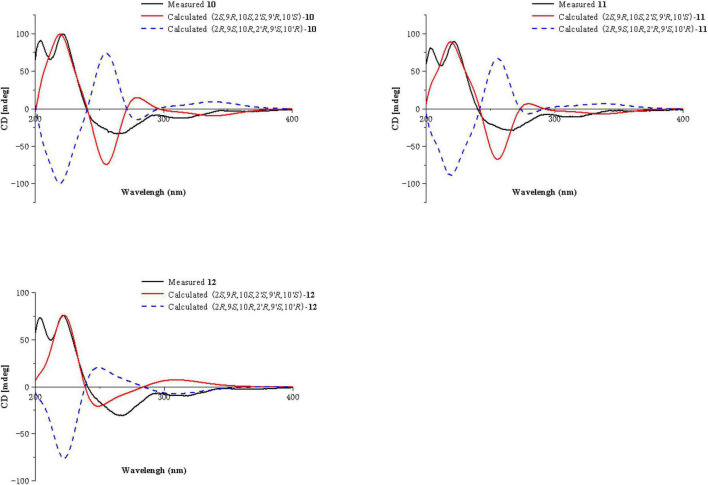
ECD calculations of **10**–**12**.

Compounds **10** and **11** were obtained as pale-yellow gum. The molecular formula of **10** was determined to be C_33_H_34_O_15_ by HRESIMS at *m/z* 671.2001 [M + H]^+^ (calcd for C_33_H_35_O_15_^+^, 671.1976), with 17 degrees of unsaturation. The molecular formula of **11** was determined to be C_34_H_36_O_15_ by HRESIMS at *m/z* 707.1934 [M + Na]^+^ (calcd for C_34_H_36_O_15_Na^+^, 707.1952), also with 17 degrees of unsaturation. By comparing the ^1^H and ^13^C NMR spectroscopic data of Compounds **9**-**11** (Table 4), it was found that the data of these three compounds were highly similar, with the only difference being that Compound **10** had one additional methoxy group compared to **9**, while Compound **11** had one additional ethoxy group compared to **9**. The key HMBC correlations (Figure 2) from H-10′ (δ_H_ 2.35)/H-11′ (δ_H_ 2.58, 2.38)/H-16′ (δ_H_ 3.68) to C-12′ (δ_C_ 173.3) indicated that the carboxylic acid group at C-12′ was methyl-esterified in Compound **10**. The key HMBC correlations (Figure 2) from H-10′ (δ_H_ 2.39)/H-11′ (δ_H_ 2.58, 2.36)/H-16′ (δ_H_ 4.16) C-12′ (δ_C_ 172.9) and from H-17′ (δ_H_ 1.28) to C-16′ (δ_C_ 60.9), in combination with the key ^1^H-^1^H COSY correlations (Figure 2) between H-16′ (δ_H_ 4.16) and H-17′ (δ_H_ 1.28) indicated that the carboxylic acid group at C-12′ was ethyl-esterified in Compound **11**. Combining the chemical shifts and coupling constants of Compound **9**, it was deduced that the relative configurations of Compounds **10** and **11** were 2S*,9R*,10S*,2′S*,9′R*,10′S*. Finally, by ECD calculations (Figure 6), the absolute configurations of Compounds **10** and **11** were determined to be 2*S*,9*R*,10*S*,2′*S*,9′*R*,10′*S*. Therefore, Compounds **10** and **11** were named paecilin N and paecilin O, respectively.

Compound **12** was obtained as a pale-yellow gum, and it possessed the molecular formula of C_34_H_38_O_16_, as indicated by the HRESIMS analysis at *m/z* 725.2049 [M + Na]^+^ (calcd for C_34_H_38_O_16_Na, 725.2058). By comparing the 1D spectroscopic data of Compound **12** (Table 3) with those of Compound **8** (Table 3), it was suggested that Compound **12** was highly similar to Compound **8**. The only difference was that Compound **12** has two additional methoxyl groups compared to Compound **8**. Considering the reduction of two degrees of unsaturation, it was assumed that both γ-butyrolactone groups of **12** were opened. This conclusion was established by the key HMBC correlations (Figure 2) from H-10/H-10′ (δ_H_ 2.37), H-11/H-11′ (δ_H_ 2.60, 2.40), and H-16/H-16′ (δ_H_ 3.70) to C-12/C-12′ (δ_C_ 173.3). By comparison with the chemical shifts and coupling constants of the ring-opening monomer of Compound **10**, it was deduced that the relative configuration of Compound **12** was 2S*,9R*,10S*,2′S*,9′R*,10′S*. Finally, the absolute configuration of Compound **12** was determined to be 2*S*,9*R*,10*S*,2′*S*,9′*R*,10′*S* by ECD calculations (Figure 6). In summary, Compound **12** was named paecilin P.

The other known analogs isolated in this study were identified as versixanthone F (**13**) (Wu et al., 2015), versixanthone A (**14**) (Wu et al., 2015), and versixanthone E (**15**) (Wu et al., 2015) by comparison of their NMR data with those reported in the literature.

### Antimicrobial activities

Compound **1** and new compounds (**2**–**12**) were evaluated for their antimicrobial activities against the bacteria *E. coli* and *S. enteritidis* and the fungus *C. albicans*. As a result, Compound **1** showed significant inhibitory activity against *C. albicans*, with an MIC of 16 μg/mL, while Compounds **8** and **10** showed inhibitory activity against the gram-negative bacterium *E. coli*, with the same MIC value of 16 μg/mL (Table 5).

**TABLE 5:** Antimicrobial activity from Compounds **1**–**12** (MIC, μg/mL).

Compounds	*Escherichia coli* ATCC 25922	*Salmonella enteritidis* ATCC 25923	*Candida albicans* ATCC 10231
**1**	64	64	16
**2**	64	128	64
**3**	256	> 512	64
**4**	> 512	>512	> 512
**5**	>512	> 512	>512
**6**	256	256	256
**7**	128	256	> 512
**8**	16	32	32
**9**	256	128	> 512
**10**	16	32	32
**11**	128	128	> 512
**12**	256	128	64
Chloramphenicol^a^	1	–	–
Penicillin^a^	–	0.78	–
Fluconazole^a^	–	–	5

^a^Positive controls.

## Conclusion

In conclusion, a total of 15 compounds, including 11 new compounds, were identified from the endophytic fungus *Xylaria curta* E10. The absolute configurations of Compounds **1**, **2,** and **6** were determined by single-crystal X-ray diffraction analysis. The absolute configurations of **3**, **4**, **5**, **7**, **8**, **9**, **10**, **11,** and **12** were determined by ECD calculations and referred to the NMR data with their structural analogs. In the antimicrobial activity assays, Compound **1** showed antifungal activity against *C. albicans*, and Compounds **8** and **10** showed antibacterial activity against *Escherichia coli*.

## Data availability statement

The original contributions presented in this study are included in the article/Supplementary material, further inquiries can be directed to the corresponding authors.

## Author contributions

H-LA isolated and provided the fungus. Z-HL and X-XL designed experiments. P-PW was responsible for compound isolation, elucidated the structures and wrote the manuscript. P-PW, B-BS, KY, X-YP, X-JM, and DX performed chemical calculations. XL and Z-HL were responsible for the evaluation of the immunosuppressive activity. All authors read and approved the final manuscript.
