# Supplementary material for: Paecilins F–P, new dimeric chromanones isolated from the endophytic fungus Xylaria curta E10, and structural revision of paecilin A
Source: Front Microbiol. 2022 Sep 2;13:922444. doi: 10.3389/fmicb.2022.922444 (PMC9478379; doi:10.3389/fmicb.2022.922444)
Supplement: Supplementary file 1 [file Table_1.DOCX]

Supplementary Material

**Paecilins F–P, New Dimeric Chromanones Isolated from the Endophytic Fungus** ***Xylaria curta* E10, and Structural Revision of Paecilin A**

**Pan-Pan Wei,^┴^ Hong-Lian Ai^┴^, Bao-bao Shi, Ke Ye, Xiao Lv, Xiao-Yan Pan, Xu-Jun Ma, Dan Xiao, Zheng-Hui Li*and Xin-Xiang Lei***

School of Pharmaceutical Sciences, South-Central University for Nationalities, Wuhan 430074, People’s Republic of China

* Correspondence:

Zheng-Hui Li

[lizhenghui@mail.scuec.edu.cn](mailto:lizhenghui@mail.scuec.edu.cn)

Xin-Xiang Lei

[xxlei@mail.scuec.edu.cn](mailto:xxlei@mail.scuec.edu.cn)

* Author Contributions.

^⊥^These authors contributed equally to this work.

**Keywords:** Dimeric chromanones; Endophytic fungus; *Xylaria curta* E10; Antimicrobial activities.

**Content**

**Sections S1. Supplementary of NMR, HRESIMS and CD spectra**

**S1.1 NMR, HRESIMS and CD spectra of paecilin A (1)**

**S1.2 NMR, HRESIMS and CD spectra of paecilin F (2)**

**S1.3 NMR, HRESIMS and CD spectra of paecilin G (3)**

**S1.4 NMR, HRESIMS and CD spectra of paecilin H (4)**

**S1.5 NMR, HRESIMS and CD spectra of paecilin I (5)**

**S1.6 NMR, HRESIMS and CD spectra of paecilin J (6)**

**S1.7 NMR, HRESIMS and CD spectra of paecilin K (7)**

**S1.8 NMR, HRESIMS and CD spectra of paecilin L (8)**

**S1.9 NMR, HRESIMS and CD spectra paecilin M (9)**

**S1.10 NMR, HRESIMS and CD spectra of paecilin N (10)**

**S1.11 NMR, HRESIMS and CD spectra of paecilin O (11)**

**S1.12 NMR, HRESIMS and CD spectra of paecilin P (12)**

**Sections S2. Computational details**

**S2.1 Computational details for paecilin G (3) (ECD)**

**S2.2 Computational details for paecilin H (4) (ECD)**

**S2.3 Computational details for paecilin I (5) (ECD)**

**S2.4 Computational details for paecilin K (7) (ECD)**

**S2.5 Computational details for paecilin L (8) (ECD)**

**S2.6 Computational details for paecilin M (9) (ECD)**

**S2.7 Computational details for paecilin N (10) (ECD)**

**S2.8 Computational details for paecilin O (11) (ECD)**

**S2.9 Computational details for paecilin P (12) (ECD)**

**Sections S3. Fungal Strain Identification**

**Sections S1. Supplementary of NMR, HRESIMS and CD spectra**

**S1.1 NMR, HRESIMS and CD spectra of paecilin A (1)**

**^1^H NMR spectrum**

**^13^C NMR and DEPT** **spectrum**

**HSQC** **spectrum**

**HMBC** **spectrum**

**^1^H-^1^H COSY** **spectrum**

**ROESY** **spectrum**

**
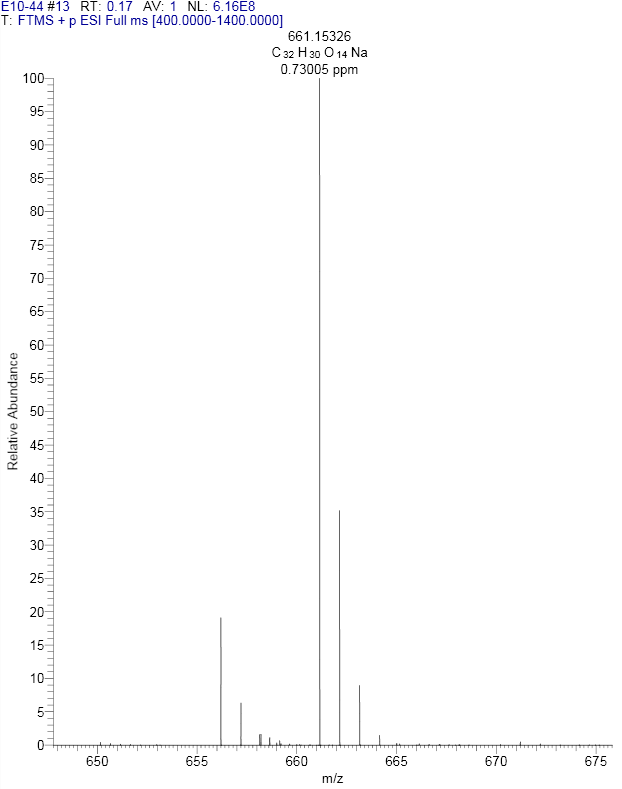
**

**HRESIMS**


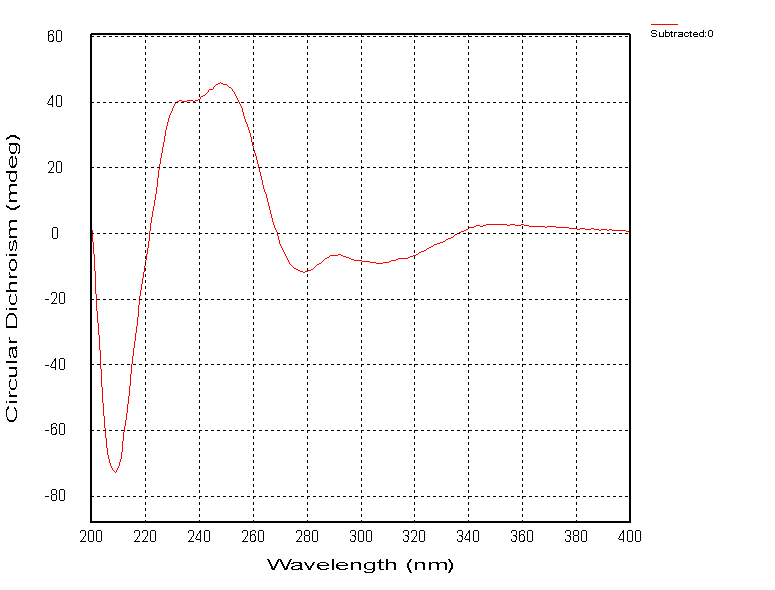


**CD spectrum**

**S1.2 NMR, HRESIMS and CD spectra of paecilin F (2)**

**^1^H NMR** **spectrum**

**^13^C NMR and DEPT** **spectrum**

**HSQC** **spectrum**

**HMBC** **spectrum**

**^1^H-^1^H COSY** **spectrum**

**ROESY** **spectrum**

**
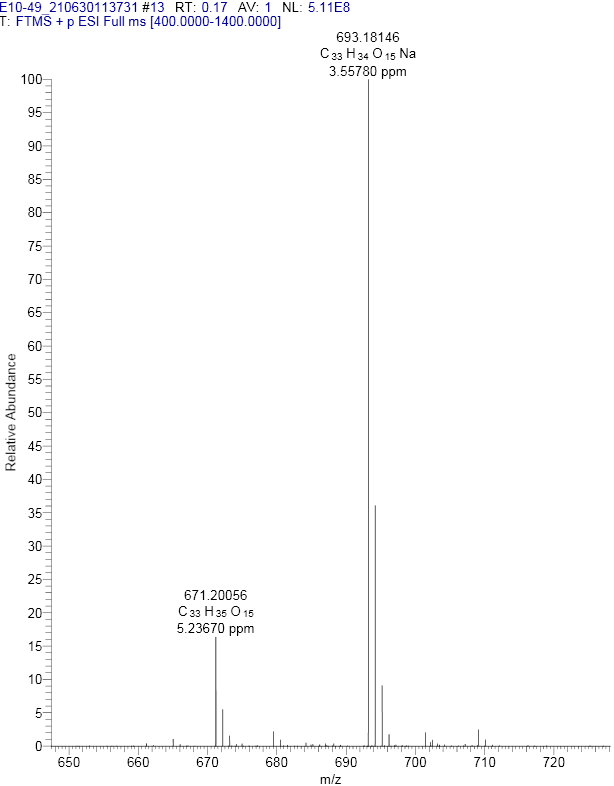
**

**HRESIMS**


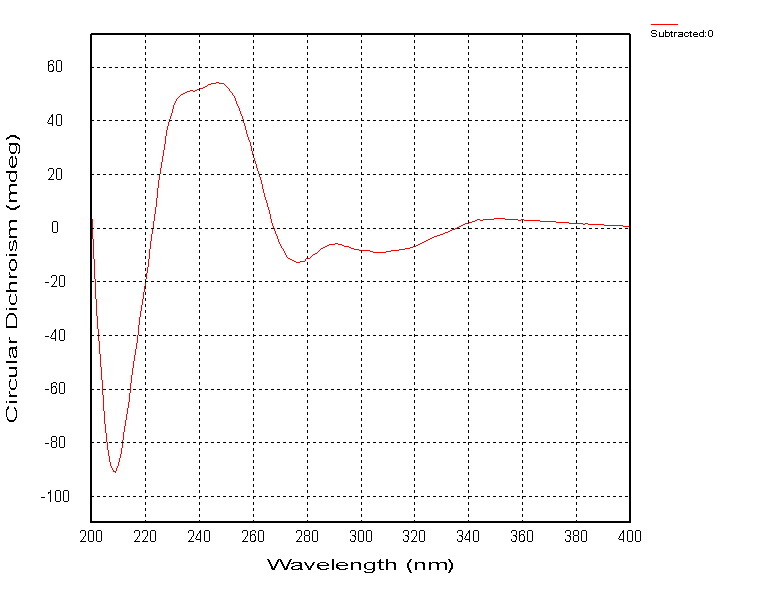


**CD spectrum**

**S1.3 NMR, HRESIMS and CD spectra of paecilin G (3)**

**^1^H NMR spectrum**

**^13^C NMR and DEPT** **spectrum**

**HSQC** **spectrum**

**HMBC** **spectrum**

**^1^H-^1^H COSY** **spectrum**

**ROESY** **spectrum**

**
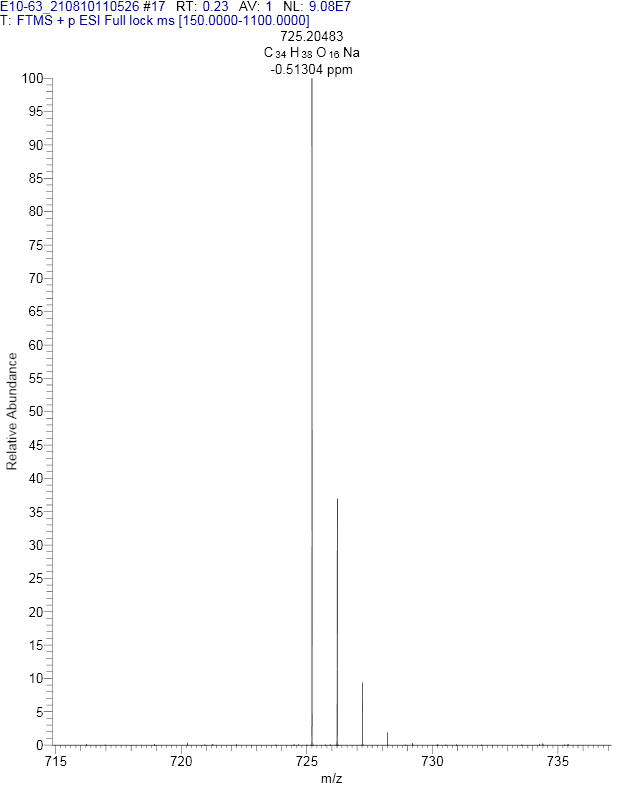
**

**HRESIMS**


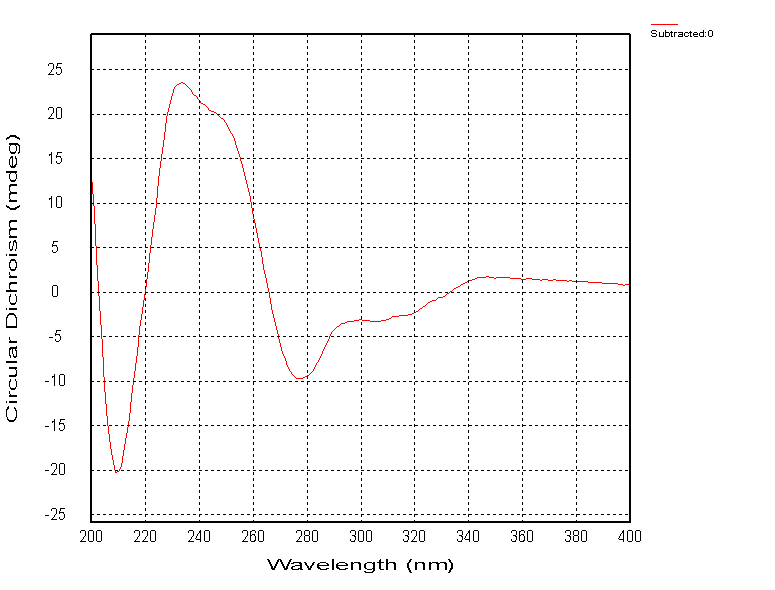


**CD spectrum**

**S1.4 NMR, HRESIMS and CD spectra of paecilin H (4)**

**^1^H NMR** **spectrum**

**^13^C NMR and DEPT** **spectrum**

**HSQC** **spectrum**

**HMBC** **spectrum**

**^1^H-^1^H COSY** **spectrum**

**ROESY** **spectrum**

**
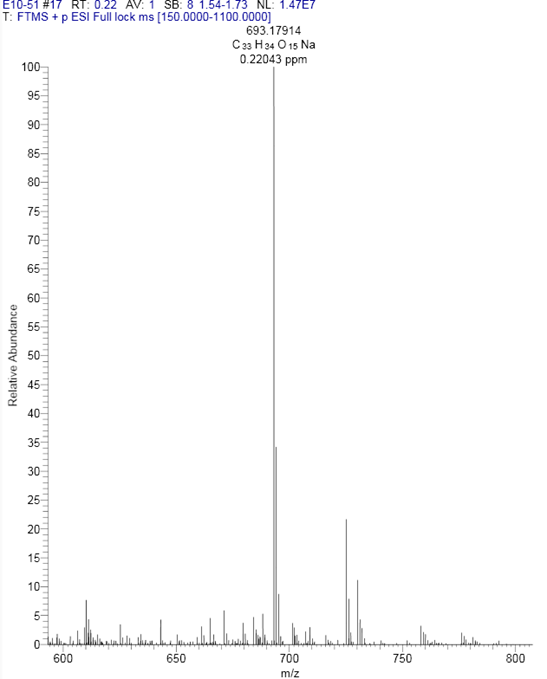
**

**HRESIMS**


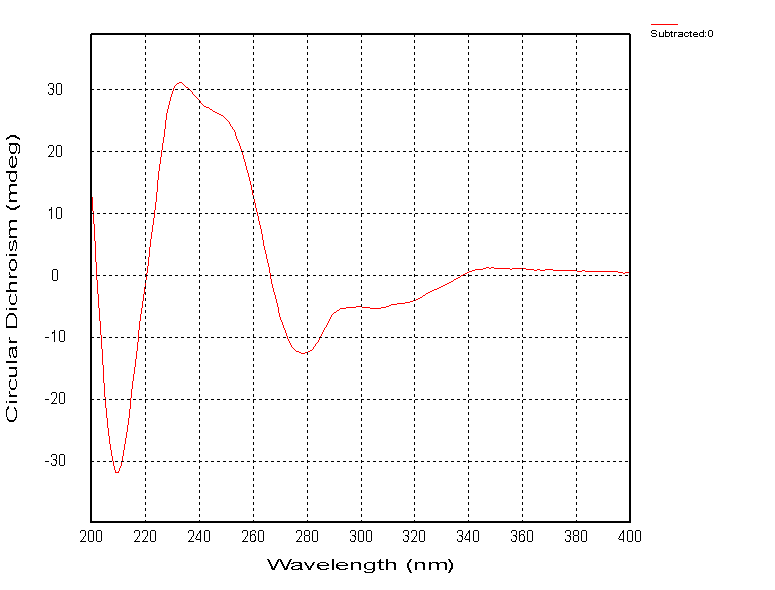


**CD spectrum**

**S1.5 NMR, HRESIMS and CD spectra of paecilin I (5)**

**^1^H NMR** **spectrum**

**^13^C NMR and DEPT** **spectrum**

**HSQC** **spectrum**

**HMBC** **spectrum**

**^1^H-^1^H COSY** **spectrum**

**ROESY** **spectrum**

**
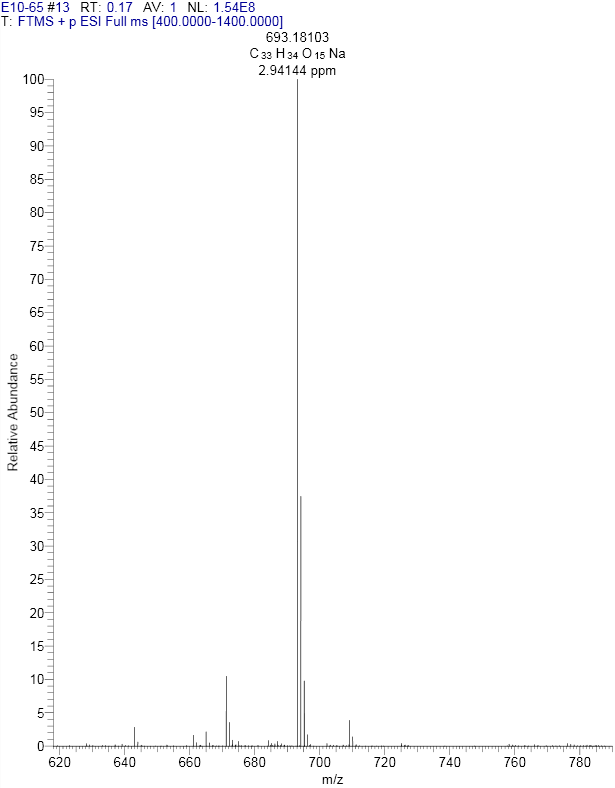
**

**HRESIMS**


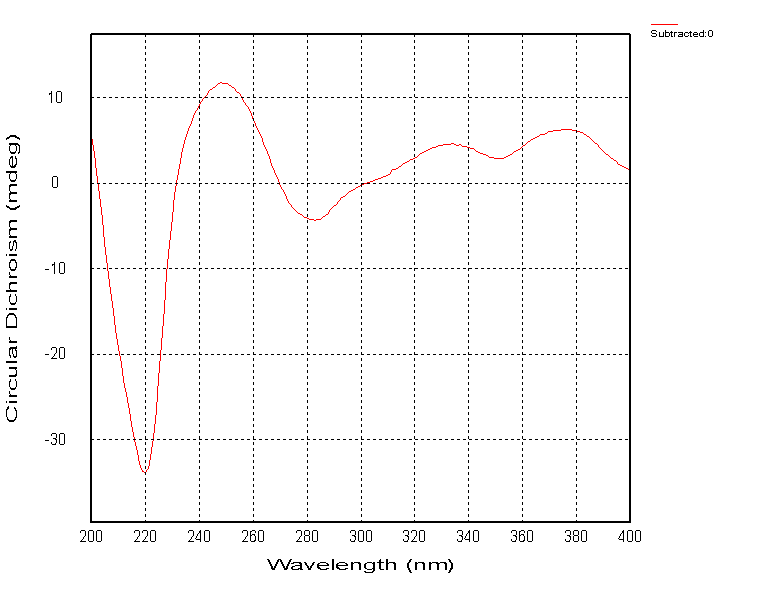


**CD spectrum**

**S1.6 NMR, HRESIMS and CD spectra of paecilin J (6)**

**^1^H NMR** **spectrum**

**^13^C NMR and DEPT** **spectrum**

**HSQC** **spectrum**

**HMBC** **spectrum**

**^1^H-^1^H COSY** **spectrum**

**ROESY** **spectrum**

**
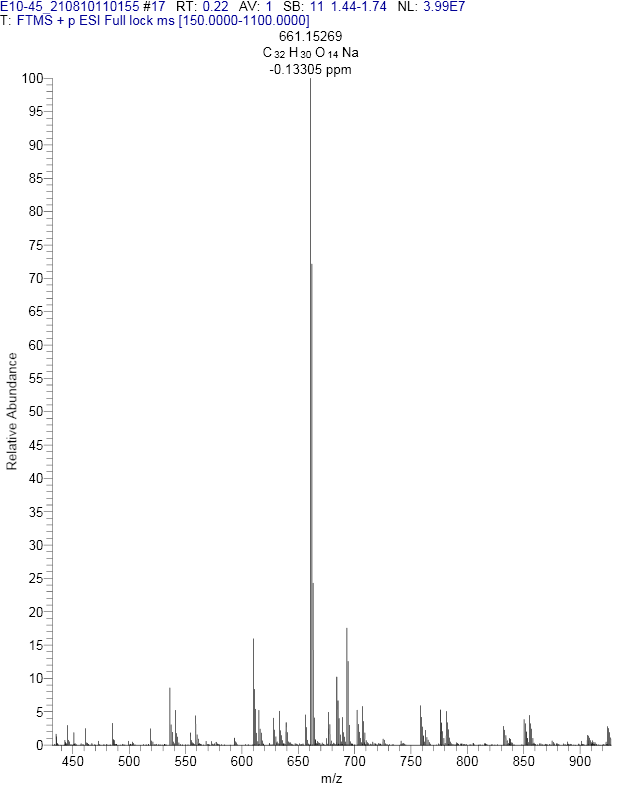
**

**HRESIMS**


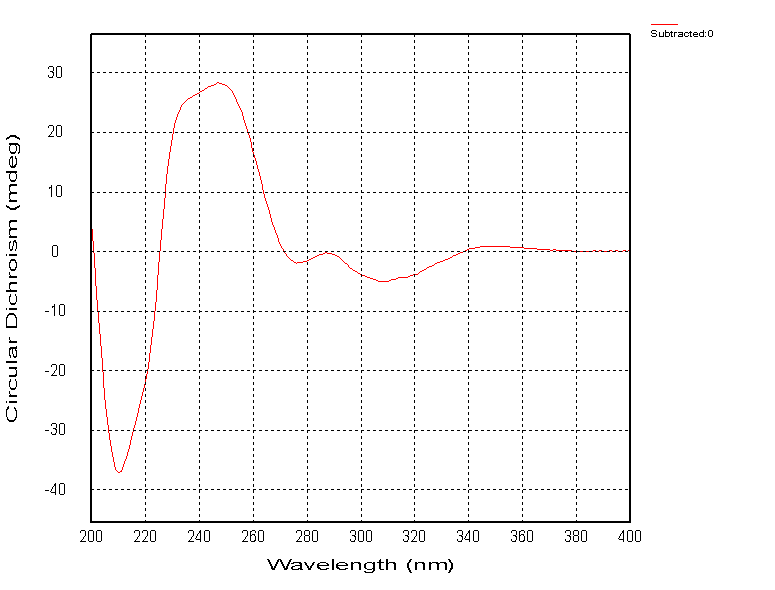


**CD spectrum**

**S1.7 NMR, HRESIMS and CD spectra of paecilin K (7)**

**^1^H NMR** **spectrum**

**^13^C NMR and DEPT** **spectrum**

**HSQC** **spectrum**

**HMBC** **spectrum**

**^1^H-^1^H COSY** **spectrum**

**ROESY** **spectrum**

**
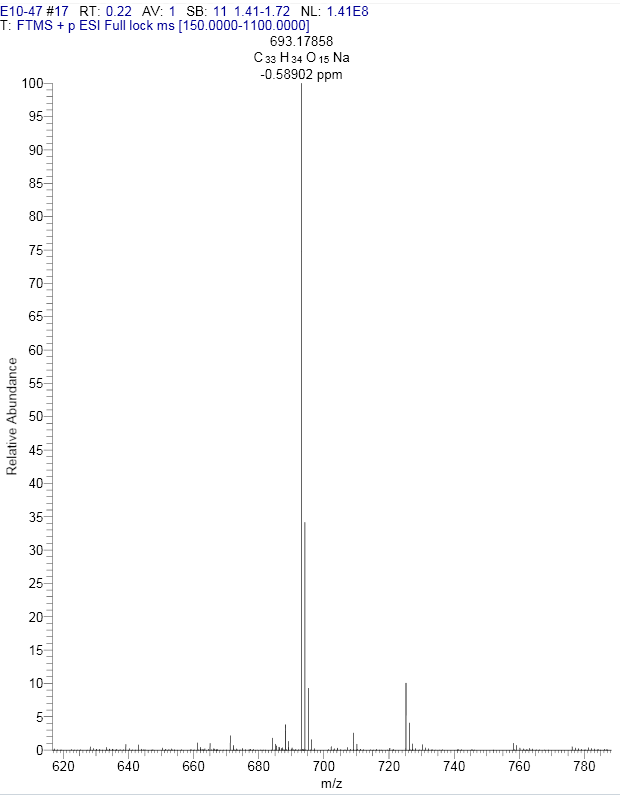
**

**HRESIMS**


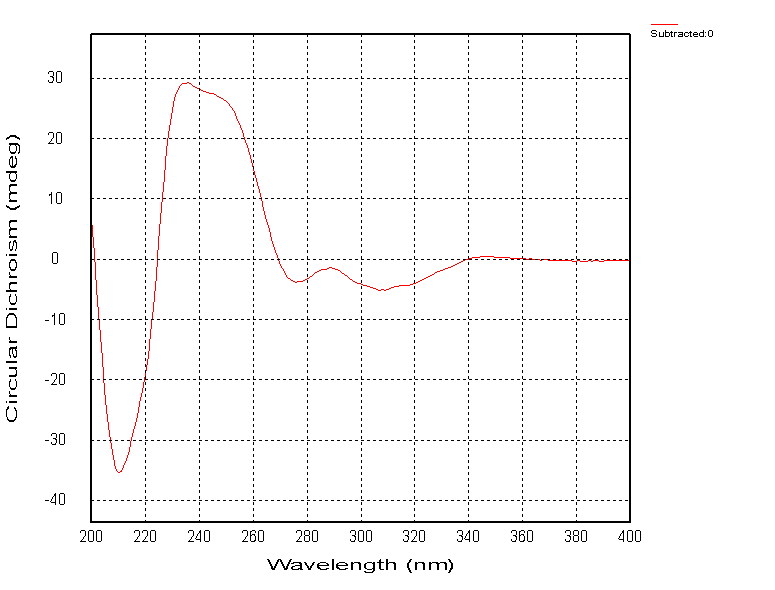


**CD spectrum**

**S1.8 NMR, HRESIMS and CD spectra of paecilin L (8)**

**^1^H NMR** **spectrum**

**^13^C NMR and DEPT** **spectrum**

**HSQC** **spectrum**

**HMBC** **spectrum**

**^1^H-^1^H COSY** **spectrum**

**ROESY** **spectrum**

**
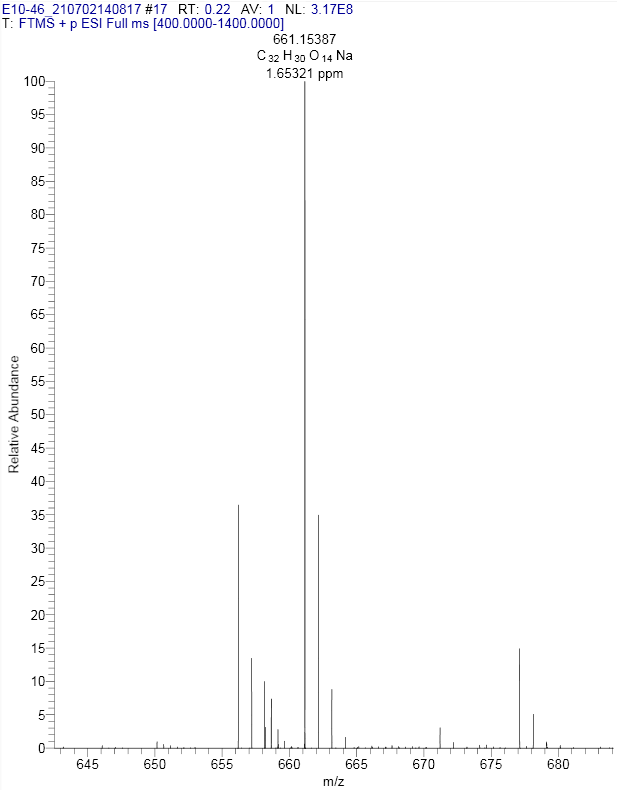
**

**HRESIMS**


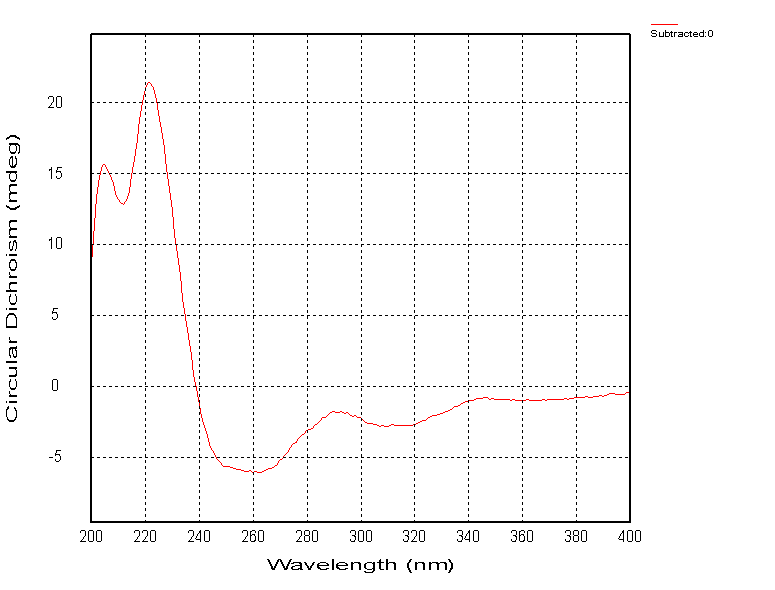


**CD spectrum**

**S1.9 NMR, HRESIMS and CD spectra of paecilin M (9)**

**^1^H NMR** **spectrum**

**^13^C NMR and DEPT** **spectrum**

**HSQC** **spectrum**

**HMBC** **spectrum**

**^1^H-^1^H COSY** **spectrum**

**ROESY** **spectrum**

**
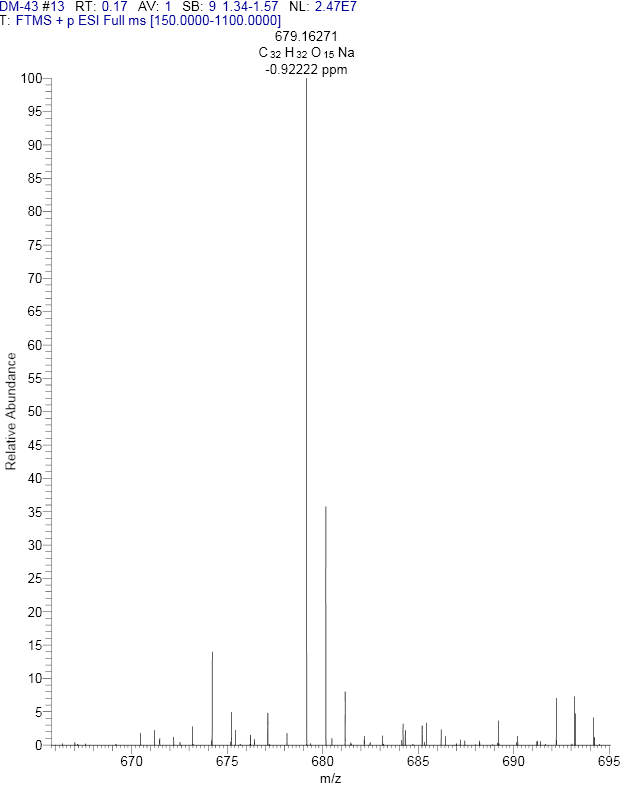
**

**HRESIMS**


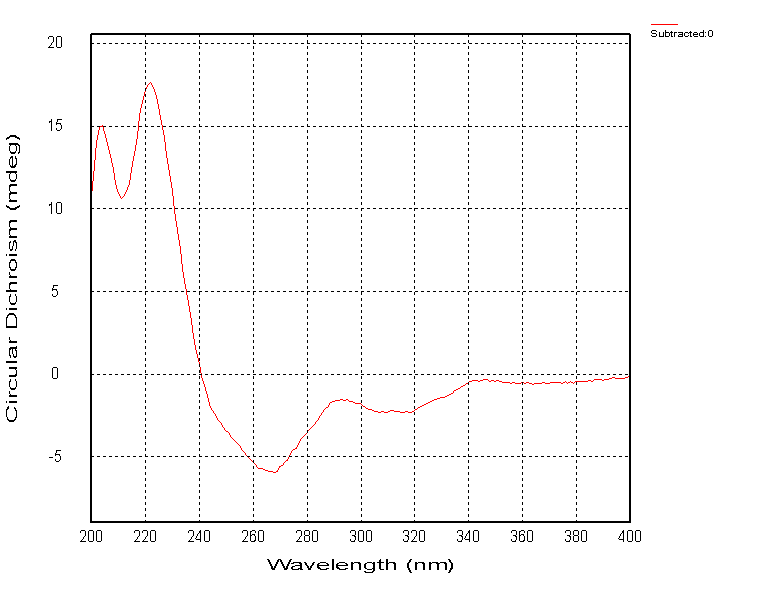


**CD spectrum**

**S1.10 NMR, HRESIMS and CD spectra of paecilin N (10)**

**^1^H NMR** **spectrum**

**^13^C NMR and DEPT** **spectrum**

**HSQC** **spectrum**

**HMBC** **spectrum**

**^1^H-^1^H COSY** **spectrum**

**ROESY** **spectrum**

**
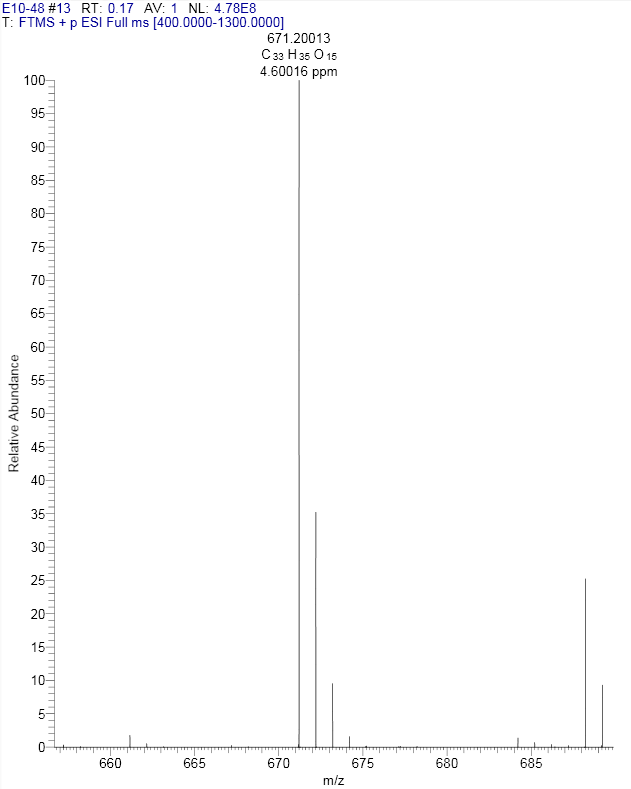
**

**HRESIMS**


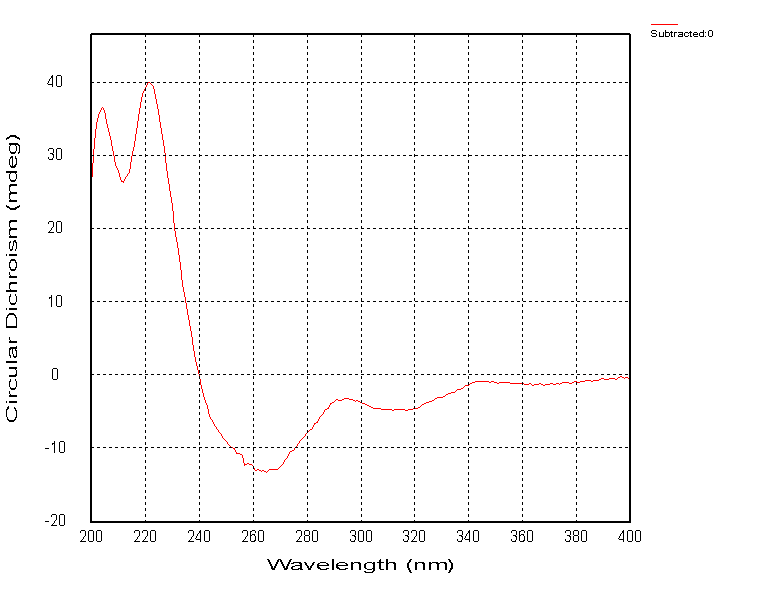


**CD spectrum**

**S1.11 NMR, HRESIMS and CD spectra of paecilin O (11)**

**^1^H NMR** **spectrum**

**^13^C NMR and DEPT** **spectrum**

**HSQC** **spectrum**

**HMBC** **spectrum**

**^1^H-^1^H COSY** **spectrum**

**ROESY** **spectrum**

**
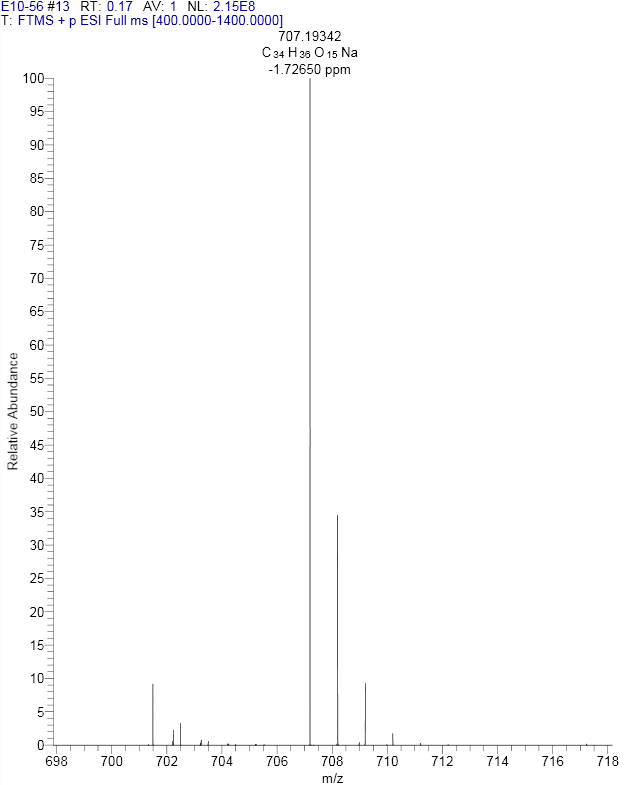
**

**HRESIMS**


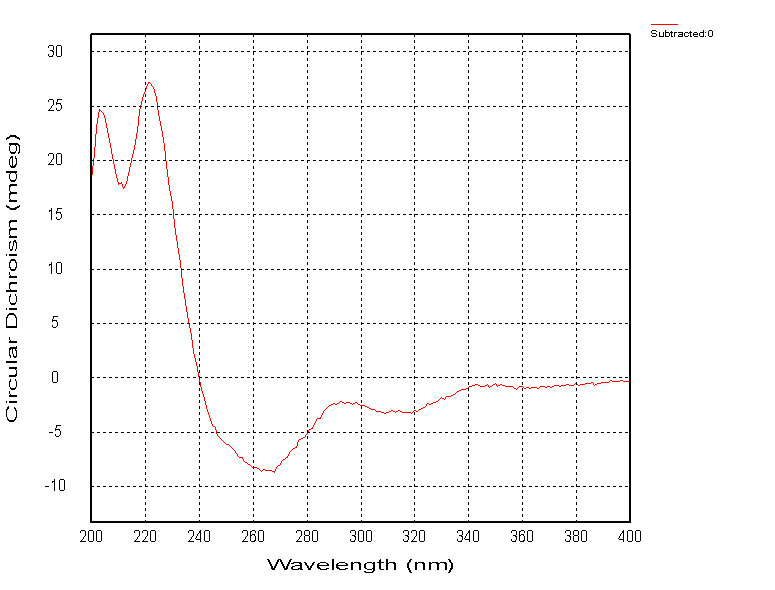


**CD spectrum**

**S1.12 NMR, HRESIMS and CD spectra of paecilin P (12)**

**^1^H NMR** **spectrum**

**^13^C NMR and DEPT** **spectrum**

**HSQC** **spectrum**

**HMBC** **spectrum**

**^1^H-^1^H COSY** **spectrum**

**ROESY** **spectrum**

**
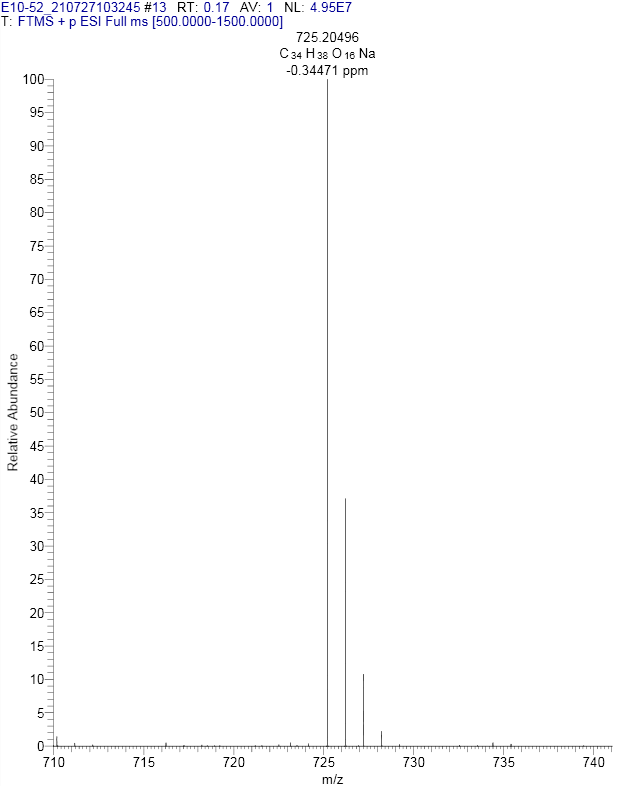
**

**HRESIMS**


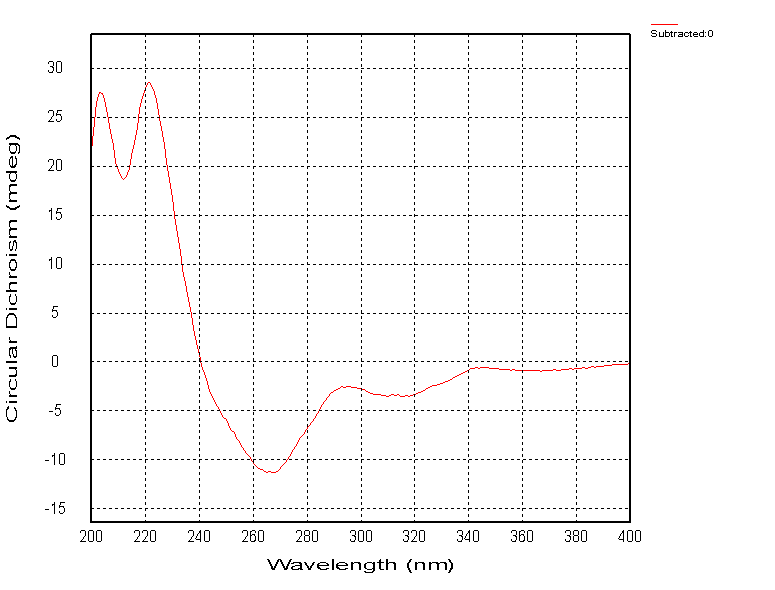


**CD spectrum**

**Sections S2. Computational details**

**S2.1 Computational details for paecilin G (3) (ECD)**

**Table S1.** Energy analysis for conformers of **3A**~**3C** at B3LYP/def2svp level in the gas phase

| *Species* | ***E′*=*E*+*ZPE*** | ***E*** | ***H*** | ***G*** | ***ΔG*** | ***ΔE*(kcal/mol)** | ***PE*%** |
| --- | --- | --- | --- | --- | --- | --- | --- |
| **3A** | -2519.636288 | -2519.587339 | -2519.586395 | -2519.72397 | 0.000000 | 1.000000 | 99.39% |
| **3B** | -2519.63431 | -2519.58545 | -2519.584505 | -2519.71903 | 0.004945 | 0.005296 | 0.53% |
| **3C** | -2519.633164 | -2519.584475 | -2519.58353 | -2519.7173 | 0.006676 | 0.000846 | 0.08% |

*E, E′, H, G*: total energy, total energy with zero point energy (*ZPE*), enthalpy, and Gibbs free energy

**Figure S1.** Optimized conformations of **3** (the relative populations are in parentheses)


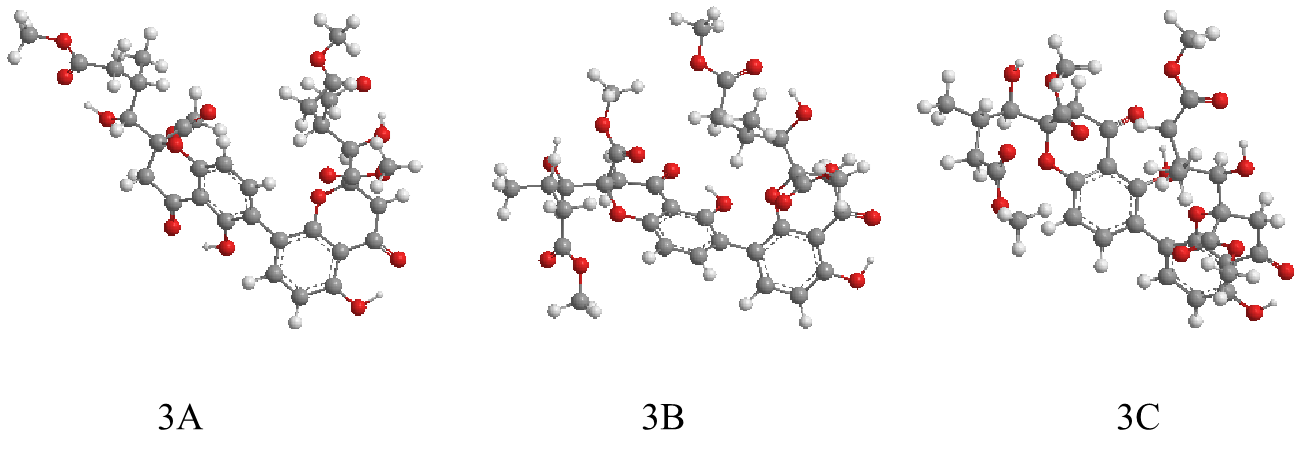


**Figure S2.** Calculated ECD spectra for **3** at the wB97xd/def2svp level in methanol with IEFPCM model (σ= 0.3 eV, UV shift 12 nm). Experimental CD spectra of **3** (black line) in MeOH.


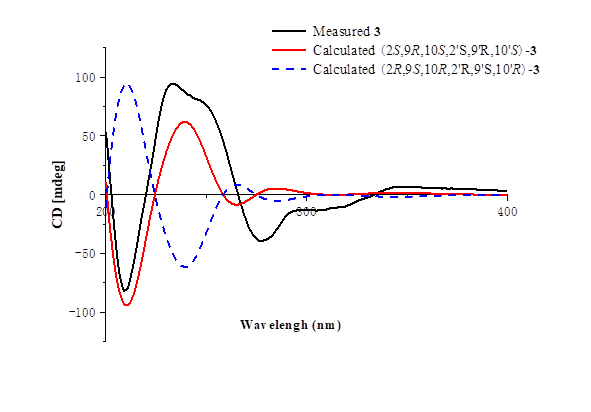


**S2.2 Computational details for paecilin H (4) (ECD)**

**Table S2.** Energy analysis for conformers of **4A**~**4C** at B3LYP/def2svp level in the gas phase

| *Species* | ***E′*=*E*+*ZPE*** | ***E*** | ***H*** | ***G*** | ***ΔG*** | ***ΔE*(kcal/mol)** | ***PE*%** |
| --- | --- | --- | --- | --- | --- | --- | --- |
| **4A** | -2404.035256 | -2403.990502 | -2403.989558 | -2404.11505 | 0.000961 | 0.361143 | 26.49% |
| **4B** | -2404.034251 | -2403.989363 | -2403.988419 | -2404.11602 | 0.000000 | 1.000000 | 73.36% |
| **4C** | -2404.030989 | -2403.986868 | -2403.985924 | -2404.11017 | 0.005848 | 0.002034 | 0.15% |

*E, E′, H, G*: total energy, total energy with zero point energy (*ZPE*), enthalpy, and Gibbs free energy

**Figure S3.** Optimized conformations of **4** (the relative populations are in parentheses)


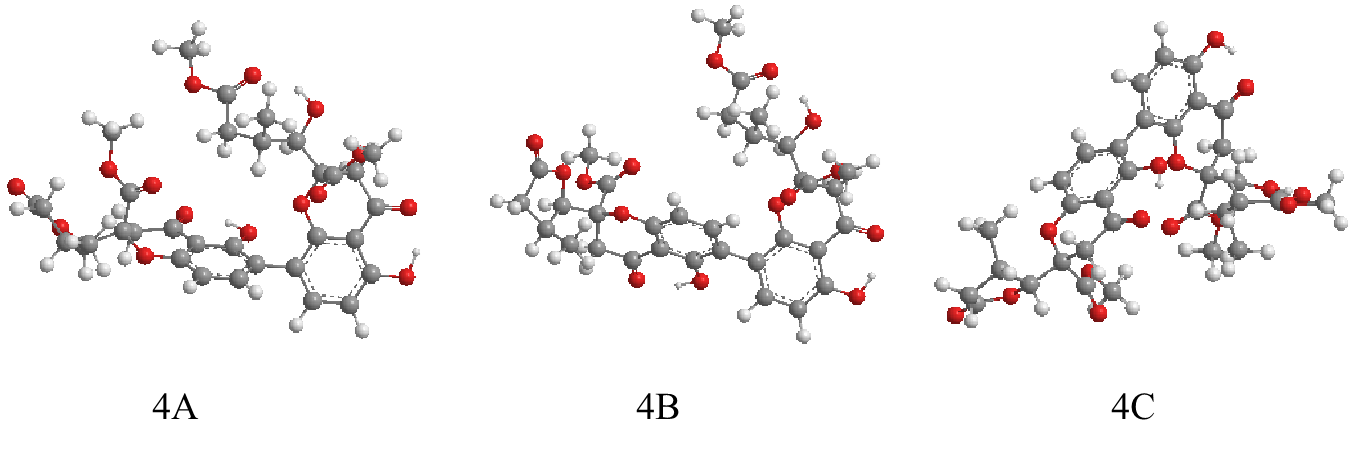


**Figure S4.** Calculated ECD spectra for **4** at the wB97xd/def2svp level in methanol with IEFPCM model (σ= 0.3 eV, UV shift 12 nm). Experimental CD spectra of **4** (black line) in MeOH.


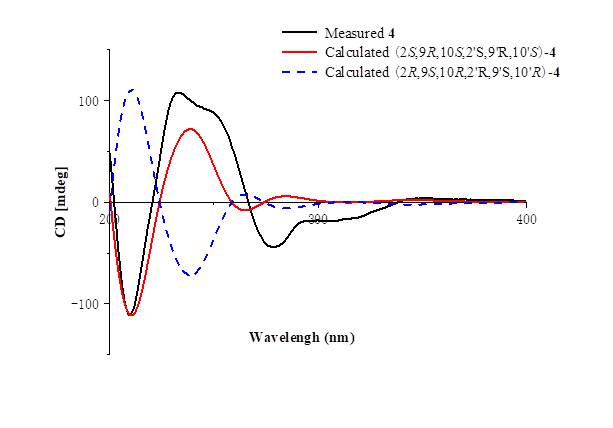


**S2.3 Computational details for paecilin I (5) (ECD)**

**Table S3.** Energy analysis for conformers of **5A**~**5P** at B3LYP/def2svp level in the gas phase

| *Species* | ***E′*=*E*+*ZPE*** | ***E*** | ***H*** | ***G*** | ***ΔG*** | ***ΔE*(kcal/mol)** | ***PE*%** |
| --- | --- | --- | --- | --- | --- | --- | --- |
| **5A** | -2404.03318 | -2403.988368 | -2403.987424 | -2404.11391 | 0.000000 | 1.000000 | 39.37% |
| **5B** | -2404.02931 | -2403.984416 | -2403.983472 | -2404.11002 | 0.003887 | 0.016253 | 0.64% |
| **5C** | -2404.031731 | -2403.986495 | -2403.985551 | -2404.11365 | 0.000257 | 0.761571 | 29.98% |
| **5D** | -2404.030291 | -2403.985023 | -2403.984079 | -2404.11193 | 0.001976 | 0.123170 | 4.85% |
| **5E** | -2404.030947 | -2403.986062 | -2403.985118 | -2404.10959 | 0.004321 | 0.010261 | 0.40% |
| **5F** | -2404.032348 | -2403.987512 | -2403.986568 | -2404.11299 | 0.000921 | 0.376782 | 14.83% |
| **5G** | -2404.031303 | -2403.986441 | -2403.985496 | -2404.11162 | 0.002284 | 0.088867 | 3.50% |
| **5H** | -2404.031303 | -2403.986441 | -2403.985496 | -2404.11162 | 0.002284 | 0.088867 | 3.50% |
| **5I** | -2404.027853 | -2403.982863 | -2403.981919 | -2404.10794 | 0.005962 | 0.001802 | 0.07% |
| **5J** | -2404.023235 | -2403.977933 | -2403.976989 | -2404.10456 | 0.009342 | 0.000050 | 0.00% |
| **5K** | -2404.029498 | -2403.984234 | -2403.98329 | -2404.11109 | 0.002812 | 0.050783 | 2.00% |
| **5L** | -2404.030769 | -2403.986333 | -2403.985389 | -2404.10805 | 0.005852 | 0.002025 | 0.08% |
| **5M** | -2404.030042 | -2403.985349 | -2403.984405 | -2404.10908 | 0.004827 | 0.006002 | 0.24% |
| **5N** | -2404.029173 | -2403.984509 | -2403.983565 | -2404.1096 | 0.004308 | 0.010403 | 0.41% |
| **5O** | -2404.028102 | -2403.983202 | -2403.982258 | -2404.10796 | 0.005942 | 0.001841 | 0.07% |
| **5P** | -2404.027156 | -2403.982136 | -2403.981191 | -2404.10765 | 0.006258 | 0.001317 | 0.05% |

*E, E′, H, G*: total energy, total energy with zero point energy (*ZPE*), enthalpy, and Gibbs free energy

**Figure S5.** Optimized conformations of **5** (the relative populations are in parentheses)


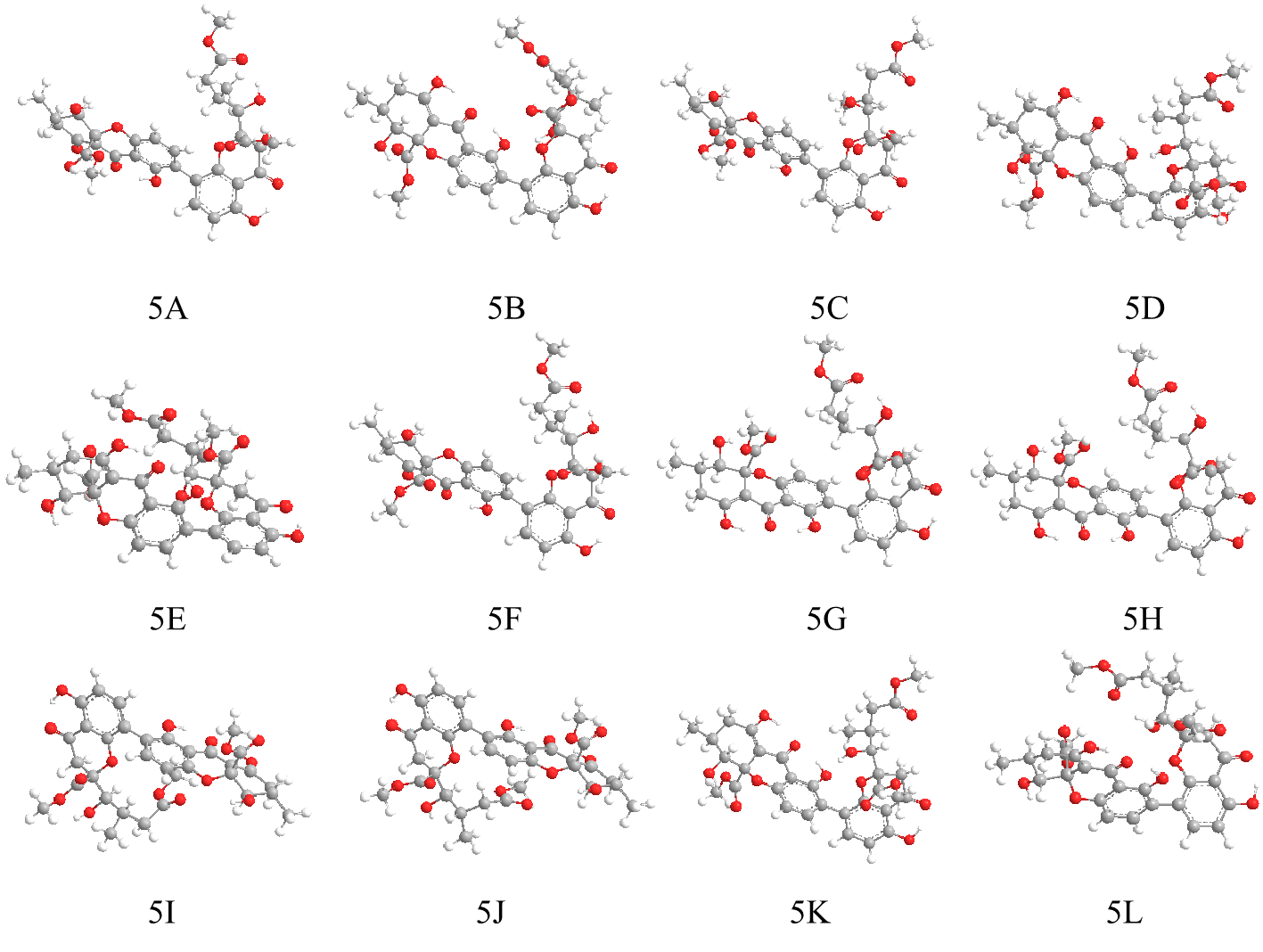


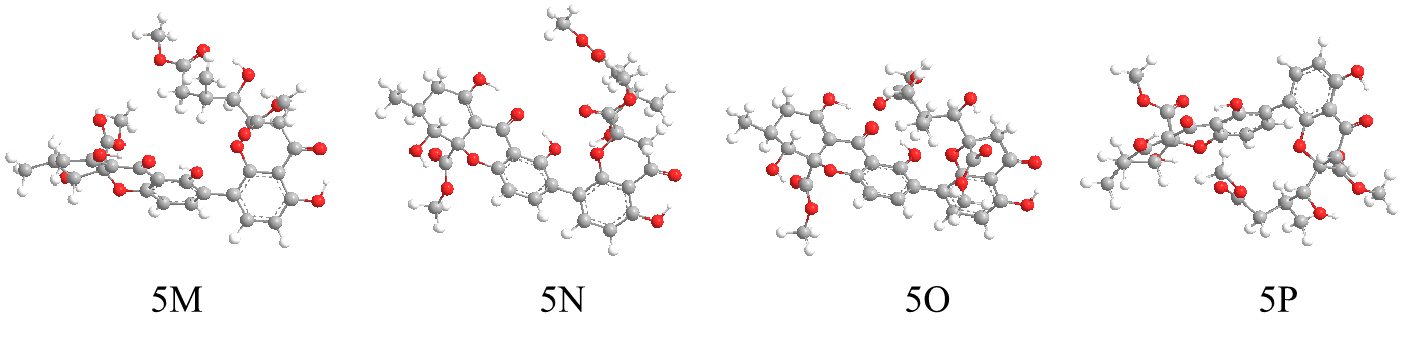


**Figure S6.** Calculated ECD spectra for **5** at the B3LYP/DGDZVP level in methanol with IEFPCM model (σ= 0.3 eV, UV shift -14 nm). Experimental CD spectra of **5** (black line) in MeOH.


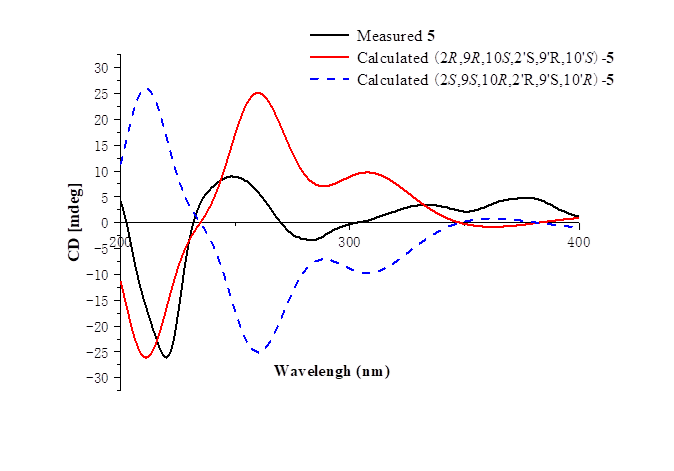


**S2.4 Computational details for paecilin K (7) (ECD)**

**Table S4.** Energy analysis for conformers of **7A**~**7I** at B3LYP/def2svp level in the gas phase

| *Species* | ***E′*=*E*+*ZPE*** | ***E*** | ***H*** | ***G*** | ***ΔG*** | ***ΔE*(kcal/mol)** | ***PE*%** |
| --- | --- | --- | --- | --- | --- | --- | --- |
| **7A** | -2404.026686 | -2403.981368 | -2403.980424 | -2404.107211 | 0.007552 | 0.000334 | 0.03% |
| **7B** | -2404.029232 | -2403.98423 | -2403.983285 | -2404.109914 | 0.004849 | 0.005863 | 0.58% |
| **7C** | -2404.028288 | -2403.983377 | -2403.982433 | -2404.108643 | 0.006120 | 0.001525 | 0.15% |
| **7D** | -2404.025415 | -2403.980083 | -2403.979139 | -2404.106967 | 0.007796 | 0.000258 | 0.03% |
| **7E** | -2404.025415 | -2403.980083 | -2403.979139 | -2404.106967 | 0.007796 | 0.000258 | 0.03% |
| **7F** | -2404.025436 | -2403.980167 | -2403.979223 | -2404.105906 | 0.008857 | 0.000084 | 0.01% |
| **7G** | -2404.028372 | -2403.98348 | -2403.982536 | -2404.109266 | 0.005497 | 0.002950 | 0.29% |
| **7H** | -2404.034196 | -2403.98916 | -2403.988216 | -2404.114763 | 0.000000 | 1.000000 | 98.83% |
| **7I** | -2404.02847 | -2403.983772 | -2403.982827 | -2404.107627 | 0.007136 | 0.000519 | 0.05% |

*E, E′, H, G*: total energy, total energy with zero point energy (*ZPE*), enthalpy, and Gibbs free energy

**Figure S7.** Optimized conformations of **7** (the relative populations are in parentheses)


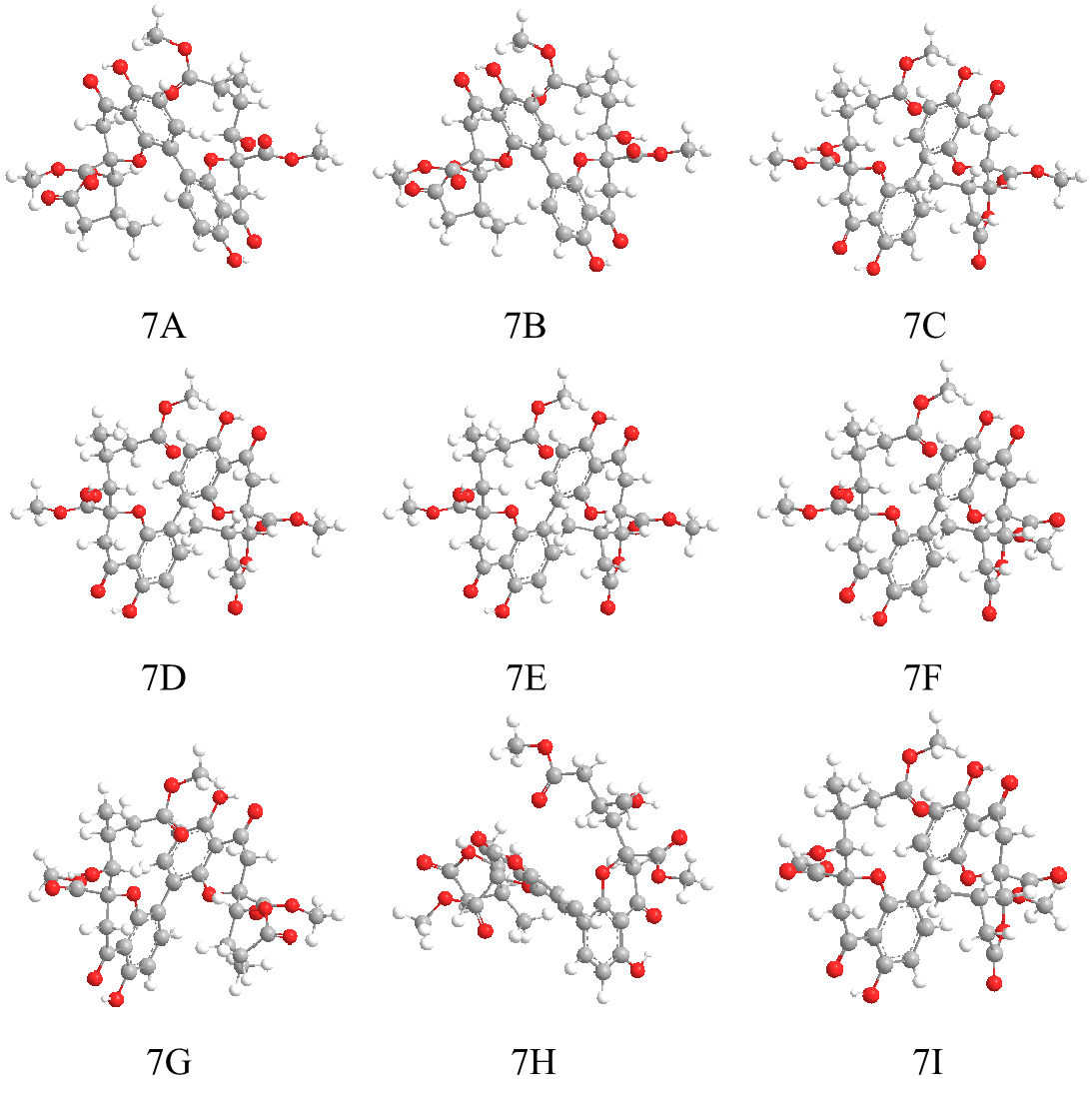


**Figure S8.** Calculated ECD spectra for **7** at the wB97xd/TZVP level in methanol with IEFPCM model (σ= 0.3 eV, UV shift 11 nm). Experimental CD spectra of **7** (black line) in MeOH.


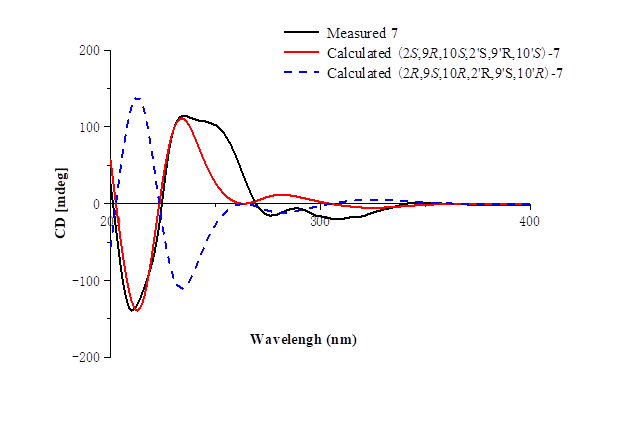


**S2.5 Computational details for paecilin L (8) (ECD)**

**Table S5.** Energy analysis for conformers of **8A**~**8N** at B3LYP/def2svp level in the gas phase

| *Species* | ***E′*=*E*+*ZPE*** | ***E*** | ***H*** | ***G*** | ***ΔG*** | ***ΔE*(kcal/mol)** | ***PE*%** |
| --- | --- | --- | --- | --- | --- | --- | --- |
| **8A** | -2288.431869 | -2288.391136 | -2288.390192 | -2288.50723 | 0.001718 | 0.161903 | 3.20% |
| **8B** | -2288.430332 | -2288.389484 | -2288.38854 | -2288.50633 | 0.002612 | 0.062773 | 1.24% |
| **8C** | -2288.431476 | -2288.39056 | -2288.389616 | -2288.50779 | 0.001153 | 0.294651 | 5.82% |
| **8D** | -2288.429568 | -2288.38869 | -2288.387745 | -2288.50581 | 0.003131 | 0.036215 | 0.72% |
| **8E** | -2288.43186 | -2288.390957 | -2288.390012 | -2288.50823 | 0.000710 | 0.471203 | 9.31% |
| **8F** | -2288.43012 | -2288.38921 | -2288.388266 | -2288.50694 | 0.002001 | 0.119949 | 2.37% |
| **8G** | -2288.431678 | -2288.390799 | -2288.389855 | -2288.50772 | 0.001226 | 0.272714 | 5.39% |
| **8H** | -2288.430774 | -2288.389818 | -2288.388874 | -2288.50725 | 0.001695 | 0.165898 | 3.28% |
| **8I** | -2288.431414 | -2288.390412 | -2288.389468 | -2288.50826 | 0.000682 | 0.485395 | 9.59% |
| **8J** | -2288.431413 | -2288.390412 | -2288.389468 | -2288.50826 | 0.000682 | 0.485395 | 9.59% |
| **8K** | -2288.433054 | -2288.392246 | -2288.391302 | -2288.50888 | 0.000068 | 0.930468 | 18.38% |
| **8L** | -2288.431489 | -2288.390576 | -2288.389632 | -2288.50812 | 0.000824 | 0.417577 | 8.25% |
| **8M** | -2288.431752 | -2288.390708 | -2288.389763 | -2288.50894 | 0.000000 | 1.000000 | 19.76% |
| **8N** | -2288.430846 | -2288.389971 | -2288.389027 | -2288.5072 | 0.001746 | 0.157169 | 3.11% |

*E, E′, H, G*: total energy, total energy with zero point energy (*ZPE*), enthalpy, and Gibbs free energy

**Figure S9.** Optimized conformations of **8** (the relative populations are in parentheses)


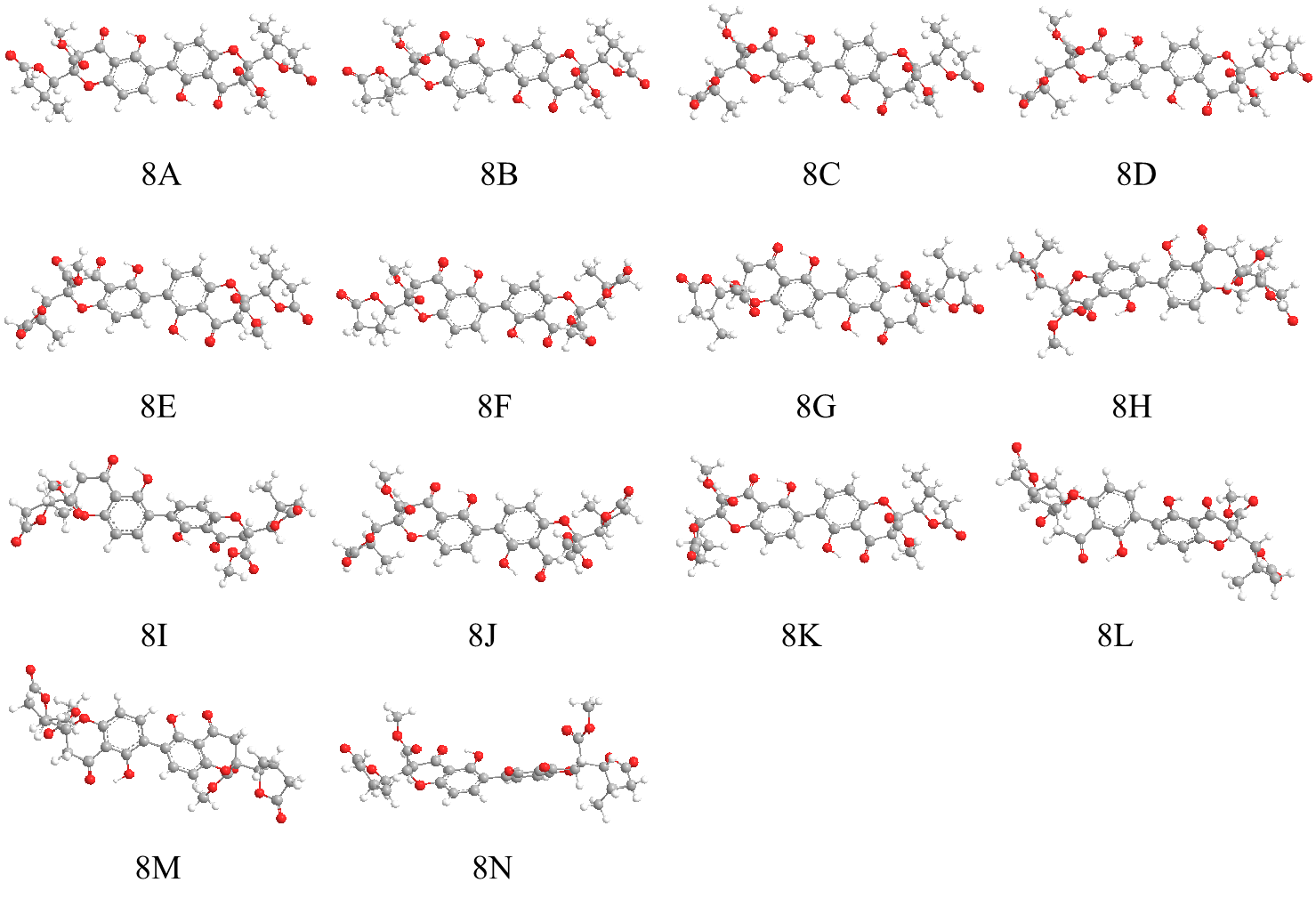


**Figure S10.** Calculated ECD spectra for **8** at the wB97xd/def2svp level in methanol with IEFPCM model (σ= 0.3 eV, UV shift 14 nm). Experimental CD spectra of **8** (black line) in MeOH.

**
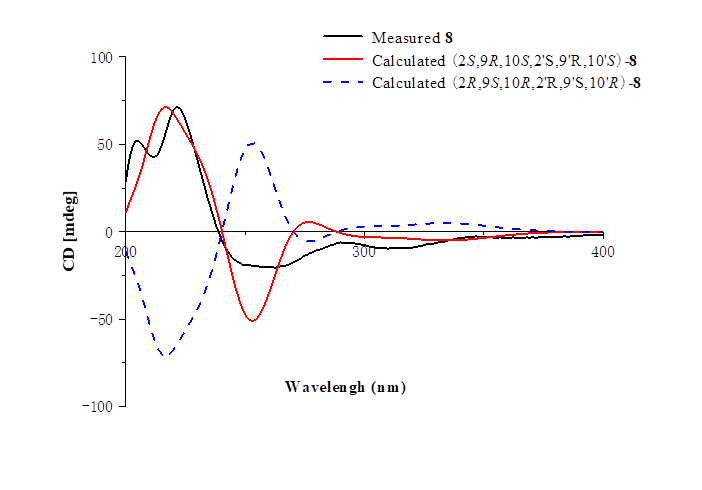
**

**S2.6 Computational details for paecilin M (9) (ECD)**

**Table S6.** Energy analysis for conformers of **9A**~**9N** at B3LYP/def2svp level in the gas phase

| *Species* | ***E′*=*E*+*ZPE*** | ***E*** | ***H*** | ***G*** | ***ΔG*** | ***ΔE*(kcal/mol)** | ***PE*%** |
| --- | --- | --- | --- | --- | --- | --- | --- |
| **9A** | -2364.781272 | -2364.737796 | -2364.736852 | -2364.86017 | 0.005668 | 0.002461 | 0.10% |
| **9B** | -2364.77956 | -2364.736022 | -2364.735078 | -2364.85861 | 0.007233 | 0.000469 | 0.02% |
| **9C** | -2364.785759 | -2364.742778 | -2364.741834 | -2364.86413 | 0.001708 | 0.163628 | 6.45% |
| **9D** | -2364.784647 | -2364.741627 | -2364.740683 | -2364.86209 | 0.003744 | 0.018912 | 0.75% |
| **9E** | -2364.780512 | -2364.737062 | -2364.736117 | -2364.85915 | 0.006693 | 0.000831 | 0.03% |
| **9F** | -2364.780903 | -2364.737449 | -2364.736505 | -2364.85979 | 0.006045 | 0.001651 | 0.07% |
| **9G** | -2364.785841 | -2364.742581 | -2364.741637 | -2364.86584 | 0.000000 | 1.000000 | 39.42% |
| **9H** | -2364.784191 | -2364.741066 | -2364.740121 | -2364.86236 | 0.003483 | 0.024939 | 0.98% |
| **9I** | -2364.783945 | -2364.740903 | -2364.739959 | -2364.86274 | 0.003098 | 0.037504 | 1.48% |
| **9J** | -2364.780576 | -2364.73716 | -2364.736216 | -2364.85927 | 0.006572 | 0.000944 | 0.04% |
| **9K** | -2364.785089 | -2364.741786 | -2364.740842 | -2364.86525 | 0.000588 | 0.536242 | 21.14% |
| **9L** | -2364.781293 | -2364.737711 | -2364.736767 | -2364.86023 | 0.005611 | 0.002615 | 0.10% |
| **9M** | -2364.785435 | -2364.742217 | -2364.741273 | -2364.86555 | 0.000285 | 0.739304 | 29.15% |
| **9N** | -2364.782256 | -2364.73906 | -2364.738116 | -2364.86116 | 0.004676 | 0.007043 | 0.28% |

*E, E′, H, G*: total energy, total energy with zero point energy (*ZPE*), enthalpy, and Gibbs free energy

**Figure S11.** Optimized conformations of **9** (the relative populations are in parentheses)


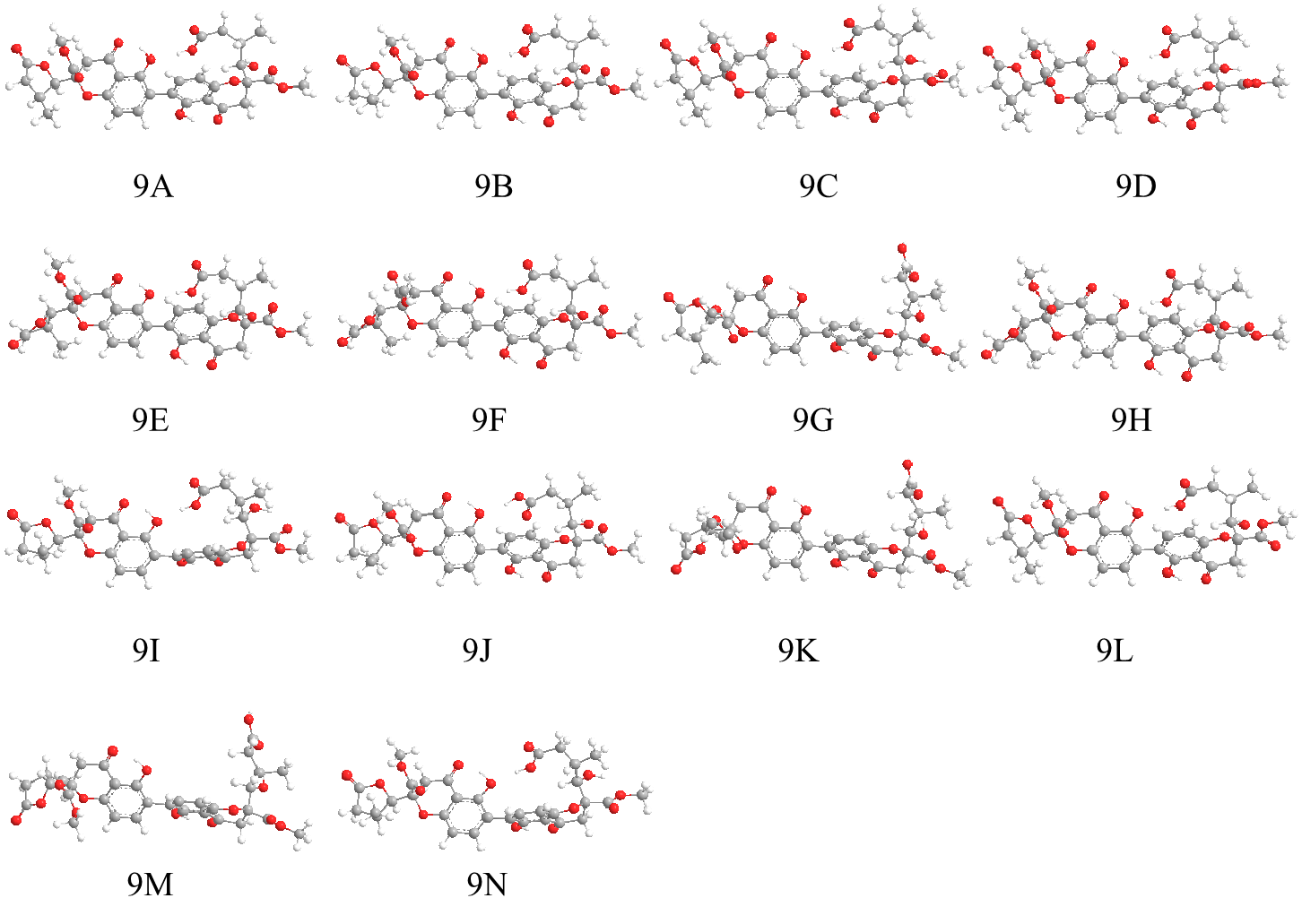


**Figure S12.** Calculated ECD spectra for **9** at the B3LYP/DGDZVP level in methanol with IEFPCM model (σ= 0.3 eV, UV shift -1 nm). Experimental CD spectra of **9** (black line) in MeOH.

**
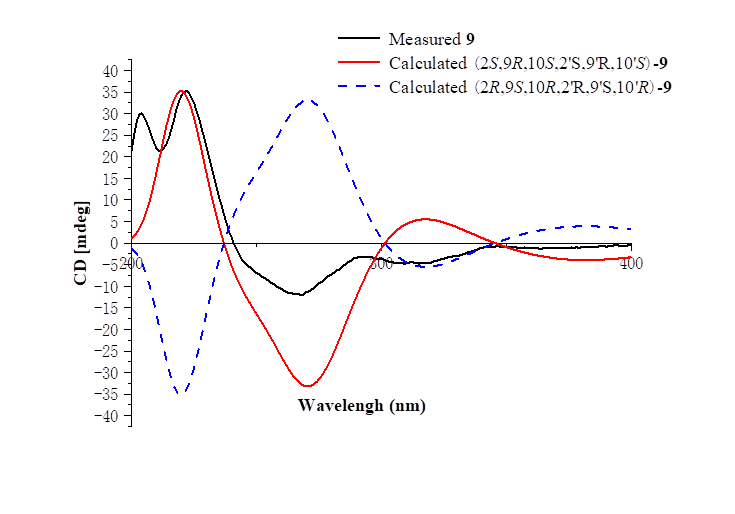
**

**S2.7 Computational details for paecilin N (10) (ECD)**

**Table S7.** Energy analysis for conformers of **10A**~**10N** at B3LYP/def2svp level in the gas phase

| *Species* | ***E′*=*E*+*ZPE*** | ***E*** | ***H*** | ***G*** | ***ΔG*** | ***ΔE*(kcal/mol)** | ***PE*%** |
| --- | --- | --- | --- | --- | --- | --- | --- |
| **10A** | -2404.03287 | -2403.987909 | -2403.986965 | -2404.11559 | 0.000678 | 0.487457 | 13.66% |
| **10B** | -2404.03341 | -2403.988463 | -2403.987519 | -2404.11627 | 0.000000 | 1.000000 | 28.03% |
| **10C** | -2404.028448 | -2403.982963 | -2403.982018 | -2404.11141 | 0.004857 | 0.005814 | 0.16% |
| **10D** | -2404.033033 | -2403.988104 | -2403.98716 | -2404.11556 | 0.000716 | 0.468216 | 13.12% |
| **10E** | -2404.027746 | -2403.982315 | -2403.981371 | -2404.11037 | 0.005901 | 0.001923 | 0.05% |
| **10F** | -2404.03232 | -2403.98721 | -2403.986266 | -2404.11442 | 0.001855 | 0.140022 | 3.92% |
| **10G** | -2404.03094 | -2403.985511 | -2403.984567 | -2404.11536 | 0.000910 | 0.381201 | 10.68% |
| **10H** | -2404.030845 | -2403.985735 | -2403.984791 | -2404.11317 | 0.003104 | 0.037267 | 1.04% |
| **10I** | -2404.031091 | -2403.986114 | -2403.98517 | -2404.1126 | 0.003671 | 0.020434 | 0.57% |
| **10J** | -2404.031405 | -2403.985987 | -2403.985043 | -2404.11526 | 0.001007 | 0.343960 | 9.64% |
| **10K** | -2404.032401 | -2403.98747 | -2403.986526 | -2404.11484 | 0.001431 | 0.219459 | 6.15% |
| **10L** | -2404.0321 | -2403.98691 | -2403.985966 | -2404.11492 | 0.001349 | 0.239384 | 6.71% |
| **10M** | -2404.03224 | -2403.987477 | -2403.986533 | -2404.1125 | 0.003771 | 0.018379 | 0.52% |
| **10N** | -2404.032504 | -2403.987642 | -2403.986698 | -2404.11477 | 0.001499 | 0.204199 | 5.72% |

*E, E′, H, G*: total energy, total energy with zero point energy (*ZPE*), enthalpy, and Gibbs free energy

**Figure S13.** Optimized conformations of **10** (the relative populations are in parentheses)


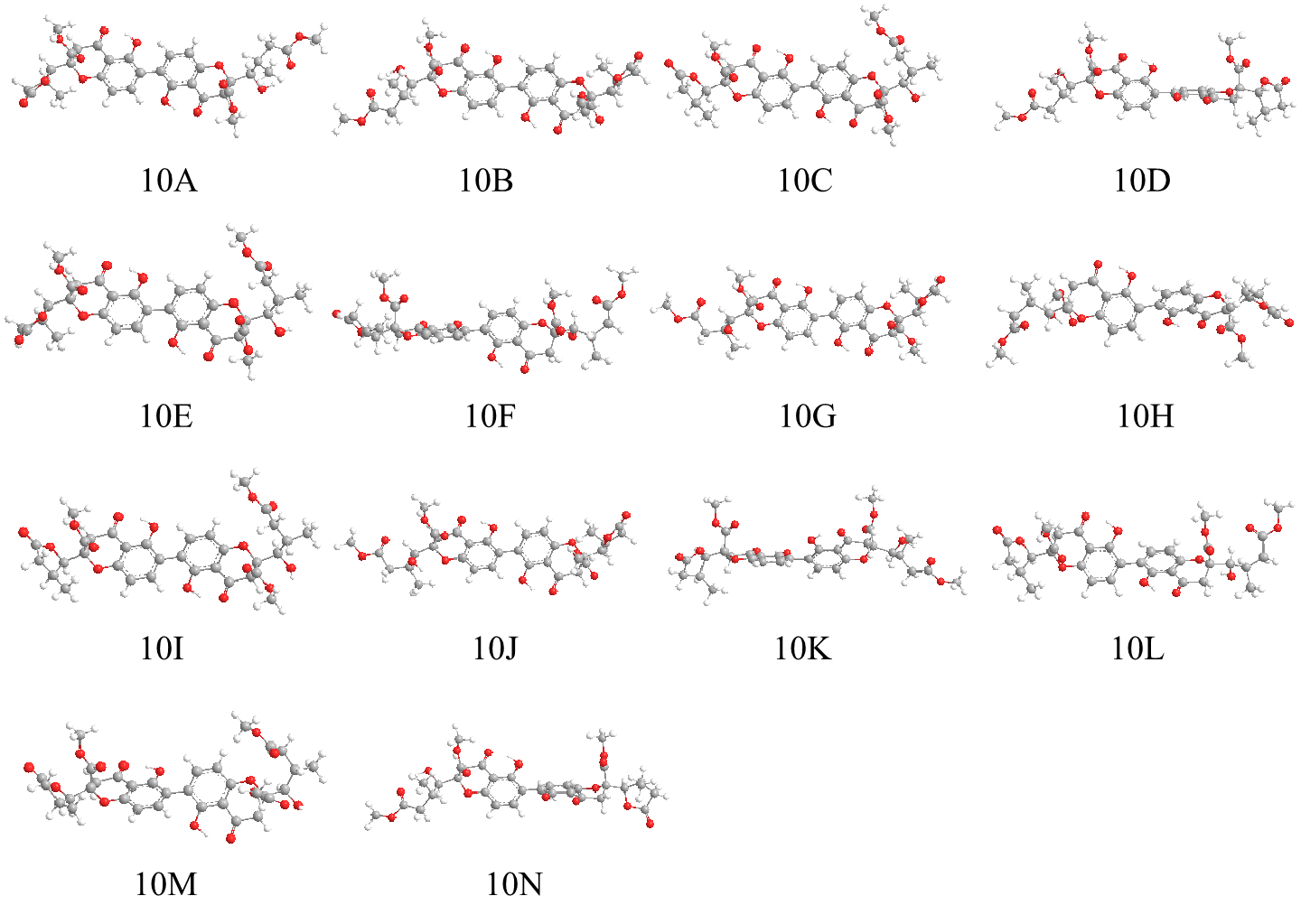


**Figure S14.** Calculated ECD spectra for **10** at the wB97xd/def2svp level in methanol with IEFPCM model (σ= 0.3 eV, UV shift 18 nm). Experimental CD spectra of **10** (black line) in MeOH.

**
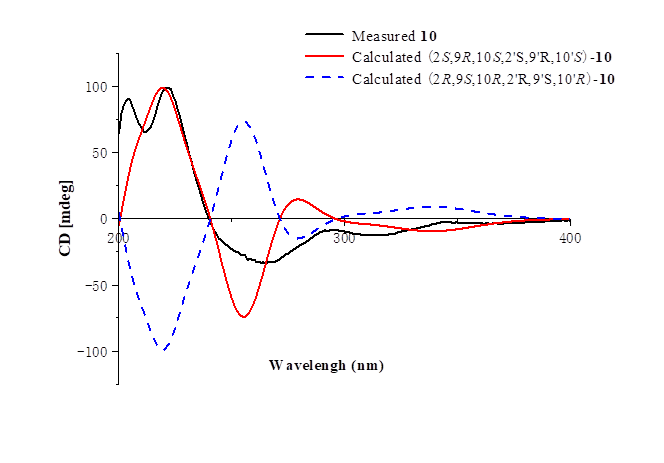
**

**S2.8 Computational details for paecilin O (11) (ECD)**

**Table S8.** Energy analysis for conformers of **11A**~**11K** at B3LYP/def2svp level in the gas phase

| *Species* | ***E′*=*E*+*ZPE*** | ***E*** | ***H*** | ***G*** | ***ΔG*** | ***ΔE*(kcal/mol)** | ***PE*%** |
| --- | --- | --- | --- | --- | --- | --- | --- |
| **11A** | -2443.296211 | -2443.250023 | -2443.249079 | -2443.38051 | 0.000601 | 0.528904 | 12.96% |
| **11B** | -2443.295558 | -2443.249286 | -2443.248342 | -2443.38043 | 0.000689 | 0.481807 | 11.81% |
| **11C** | -2443.296036 | -2443.249801 | -2443.248857 | -2443.38048 | 0.000639 | 0.508027 | 12.45% |
| **11D** | -2443.294102 | -2443.247398 | -2443.246454 | -2443.38001 | 0.001106 | 0.309700 | 7.59% |
| **11E** | -2443.295144 | -2443.248962 | -2443.248018 | -2443.37967 | 0.001441 | 0.217145 | 5.32% |
| **11F** | -2443.295812 | -2443.249464 | -2443.24852 | -2443.38111 | 0.000000 | 1.000000 | 24.51% |
| **11G** | -2443.293461 | -2443.246723 | -2443.245779 | -2443.38027 | 0.000845 | 0.408386 | 10.01% |
| **11H** | -2443.29507 | -2443.248778 | -2443.247834 | -2443.38006 | 0.001054 | 0.327246 | 8.02% |
| **11I** | -2443.293569 | -2443.246907 | -2443.245963 | -2443.37932 | 0.001797 | 0.148900 | 3.65% |
| **11J** | -2443.29327 | -2443.246653 | -2443.245709 | -2443.37922 | 0.001899 | 0.133643 | 3.28% |
| **11K** | -2443.292707 | -2443.246257 | -2443.245313 | -2443.3772 | 0.003914 | 0.015794 | 0.39% |

*E, E′, H, G*: total energy, total energy with zero point energy (*ZPE*), enthalpy, and Gibbs free energy

**Figure S15.** Optimized conformations of **11** (the relative populations are in parentheses)


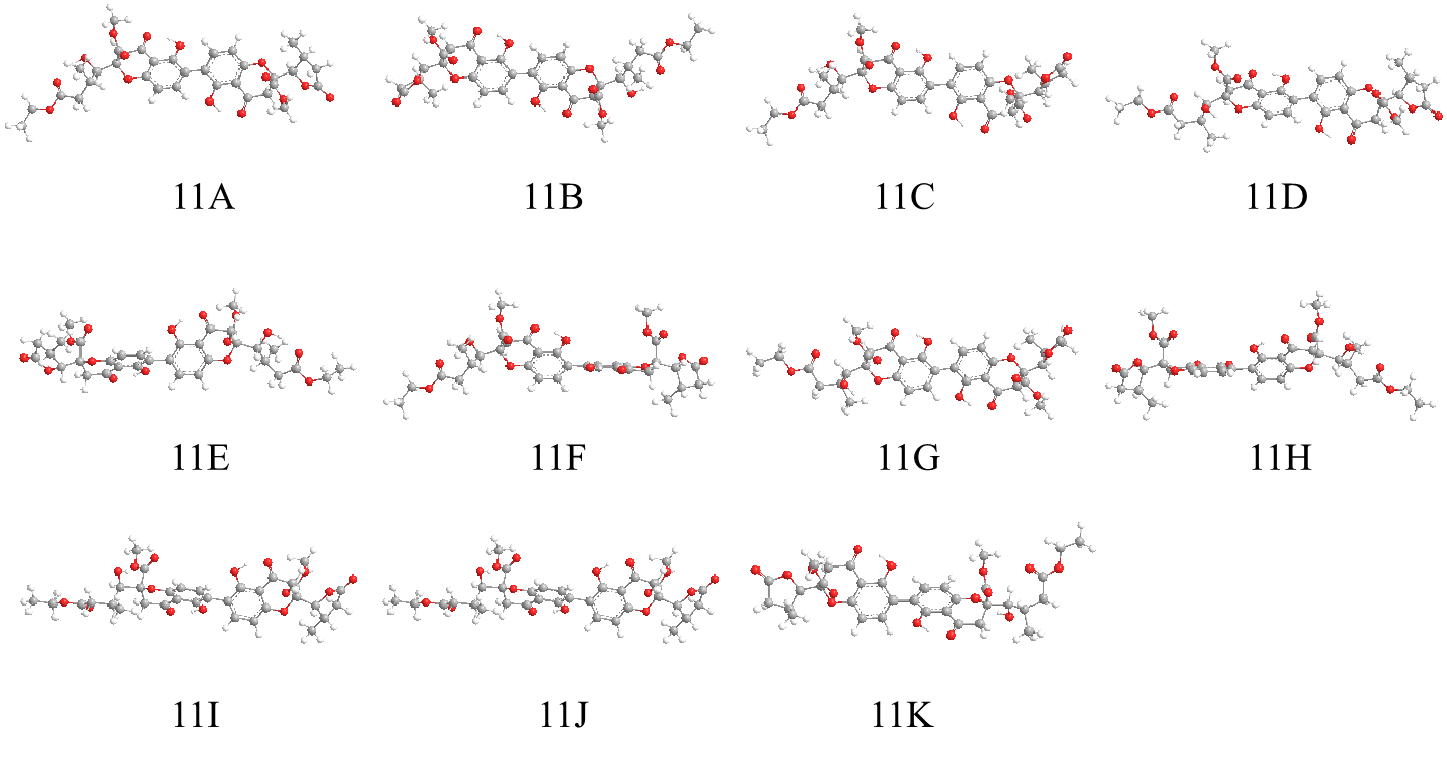


**Figure S16.** Calculated ECD spectra for **11** at the wB97xd/def2svp level in methanol with IEFPCM model (σ= 0.3 eV, UV shift 17 nm). Experimental CD spectra of **11** (black line) in MeOH.

**
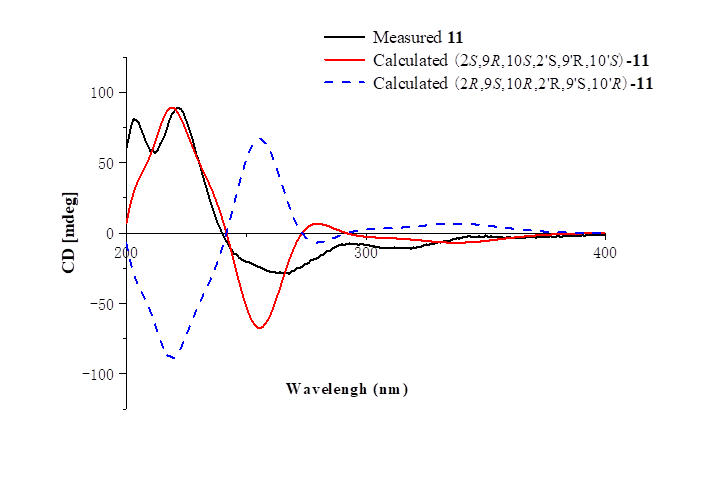
**

**S2.9 Computational details for paecilin P (12) (ECD)**

**Table S9.** Energy analysis for conformers of **12A**~**12H** at B3LYP/def2svp level in the gas phase

| *Species* | ***E′*=*E*+*ZPE*** | ***E*** | ***H*** | ***G*** | ***ΔG*** | ***ΔE*(kcal/mol)** | ***PE*%** |
| --- | --- | --- | --- | --- | --- | --- | --- |
| **12A** | -2519.627763 | -2519.577882 | -2519.576937 | -2519.71758 | 0.001966 | 0.124482 | 8.22% |
| **12B** | -2519.630623 | -2519.581128 | -2519.580184 | -2519.71954 | 0.000000 | 1.000000 | 66.02% |
| **12C** | -2519.627522 | -2519.577627 | -2519.576683 | -2519.71773 | 0.001811 | 0.146707 | 9.69% |
| **12D** | -2519.629595 | -2519.580488 | -2519.579544 | -2519.71715 | 0.002389 | 0.079508 | 5.25% |
| **12E** | -2519.628957 | -2519.579543 | -2519.578598 | -2519.71722 | 0.002319 | 0.085631 | 5.65% |
| **12F** | -2519.627277 | -2519.577408 | -2519.576463 | -2519.71675 | 0.002789 | 0.052036 | 3.44% |
| **12G** | -2519.622075 | -2519.572775 | -2519.571831 | -2519.70951 | 0.010029 | 0.000024 | 0.00% |
| **12H** | -2519.625498 | -2519.57557 | -2519.574626 | -2519.71611 | 0.003430 | 0.026380 | 1.74% |

*E, E′, H, G*: total energy, total energy with zero point energy (*ZPE*), enthalpy, and Gibbs free energy

**Figure S17.** Optimized conformations of **12** (the relative populations are in parentheses)


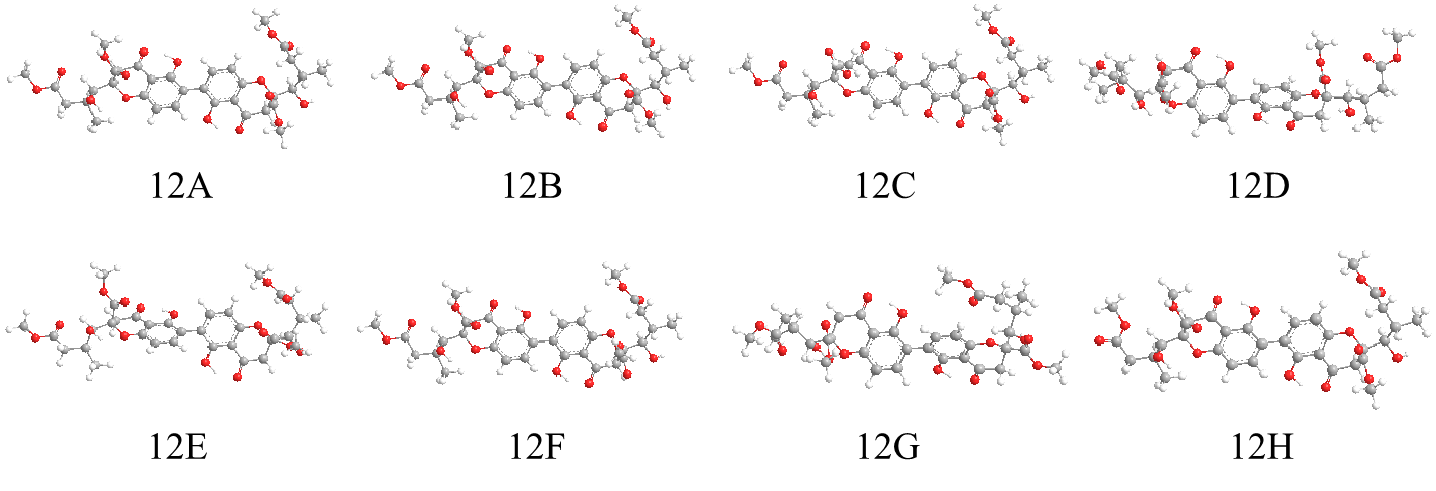


**Figure S18.** Calculated ECD spectra for **12** at the wB97xd/def2svp level in methanol with IEFPCM model (σ= 0.3 eV, UV shift 17 nm). Experimental CD spectra of **12** (black line) in MeOH.

**
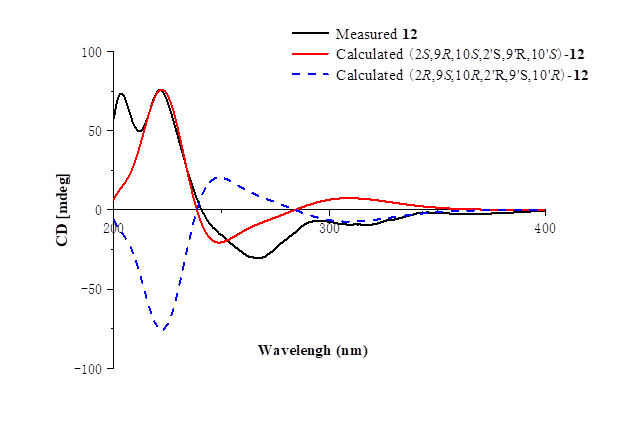
**

**Sections S3. Fungal Strain Identification**

The strain required in this study was isolated from fresh healthy potato tissues collected from Dali City, Yunnan Province, China, and identified as *Xylaria curta* E10 according to the ITS sequence (GenBank Accession No. KJ883611.1, query cover 100%, maximum identity 99%). At present, the strain is stored in the microbial seed bank of the School of Pharmacy, South-Central University for Nationalities. The fungus *Xylaria curta* E10 was fermented on solid rice medium (100 g of rice and 100 ml of water, in each 500 ml culture flask, with a total of 15 kg of rice), and was cultured at 24 °C for one month.

**
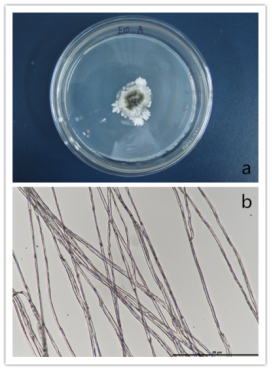
**

**Figure S19.** Morphology of colony and mycelium of *Xylaria curta* E10.
